# Hydrogen Production via Hydrolysis and Alcoholysis of Light Metal-Based Materials: A Review

**DOI:** 10.1007/s40820-021-00657-9

**Published:** 2021-06-05

**Authors:** Liuzhang Ouyang, Jun Jiang, Kang Chen, Min Zhu, Zongwen Liu

**Affiliations:** 1 School of Materials Science and Engineering, Guangdong Provincial Key Laboratory of Advanced Energy Storage Materials, South China University of Technology, Guangzhou, 510641 People’s Republic of China; 2 China-Australia Joint Laboratory for Energy and Environmental Materials, Key Laboratory of Fuel Cell Technology of Guangdong Province, Guangzhou, 510641 People’s Republic of China; 3 School of Chemical and Biomolecular Engineering, The University of Sydney, Sydney, NSW 2006 Australia; 4 The University of Sydney Nano Institute, The University of Sydney, Sydney, NSW 2006 Australia

**Keywords:** Hydrolysis, Alcoholysis, Light metal-based materials, Borohydrides, Magnesium, Aluminum, Hydrogen production

## Abstract

As an environmentally friendly and high-density energy carrier, hydrogen has been recognized as one of the ideal alternatives for fossil fuels. One of the major challenges faced by “hydrogen economy” is the development of efficient, low-cost, safe and selective hydrogen generation from chemical storage materials. In this review, we summarize the recent advances in hydrogen production via hydrolysis and alcoholysis of light-metal-based materials, such as borohydrides, Mg-based and Al-based materials, and the highly efficient regeneration of borohydrides. Unfortunately, most of these hydrolysable materials are still plagued by sluggish kinetics and low hydrogen yield. While a number of strategies including catalysis, alloying, solution modification, and ball milling have been developed to overcome these drawbacks, the high costs required for the “one-pass” utilization of hydrolysis/alcoholysis systems have ultimately made these techniques almost impossible for practical large-scale applications. Therefore, it is imperative to develop low-cost material systems based on abundant resources and effective recycling technologies of spent fuels for efficient transport, production and storage of hydrogen in a fuel cell-based hydrogen economy.

## Highlights

- An overview of the recent advances in hydrogen production from light metal-based materials is presented, including hydrolysis of Mg-based alloys and hydrides, hydrolysis of Al-based alloys and hydrides and (catalyzed) hydrolysis/alcoholysis of borohydrides.
- Hydrogen production and storage in a close loop are achieved via hydrolysis and regeneration of borohydrides, demonstrating a promising step toward the large-scale application of chemical hydrogen storage materials in a fuel cell-based hydrogen economy.

## Graphical abstract

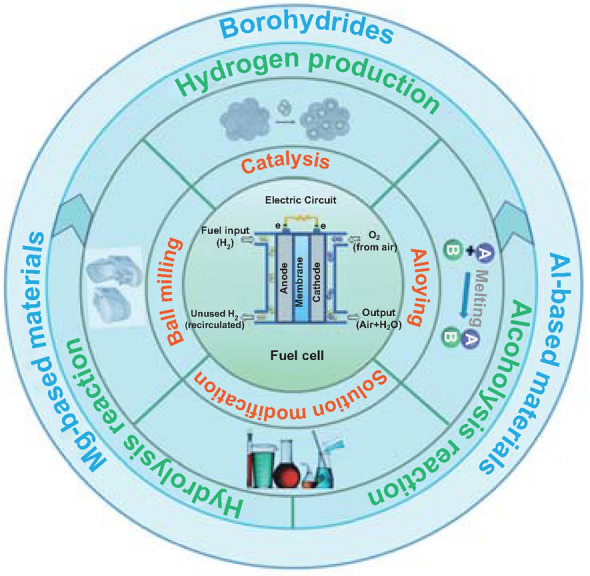

## Introduction

Hydrogen, the most abundant content in the universe, has a number of advantages over conventional fuels. It has a high energy density (142 MJ kg^−1^) and is environmentally friendly. As such, hydrogen energy economy was proposed by Hofman et al. [1] in the early 70s. Encouragingly, the emerging of proton exchange membrane fuel cells (PEMFCs) in the mid-2000s made large-scale hydrogen applications achievable in vehicles or portable electronic devices [2–4]. Particularly, a commercially available car driven by 4 kg of hydrogen fuel can run 400 km with zero carbon oxide emissions [5]. The energy efficiency of this hydrogen ‘burnt’ process via electrochemically combining with oxygen in fuel cell may reach 70% with less Carnot efficiency loss compared to that in an internal combustion engine [6]. However, the major obstacles for the advent of the hydrogen economy are the absence of efficient strategies for both hydrogen storage and production. Therefore, it is urgent to develop effective solutions to solve these problems from the view of the futuristic aspect of the utilization of hydrogen in stationary, portable and automotive applications [7–9].

As it is known, hydrogen storage methods generally are classified into three types: solid-, liquid- and gas-state. Though ultrahigh-pressure hydrogen and cryogenic-liquid hydrogen technologies are relatively mature and have been applied in various prototype vehicles [10], the hydrogen density barely meets the targets determined by the US Department of Energy (DOE) [11]. For ultrahigh-pressure hydrogen gas, the hydrogen-storage targets of DOE upon onboard hydrogen applications in terms of gravimetric and volumetric density are 1.6 and 2.1 times higher (Table 1), respectively, than the values achieved to date using common 700-bar tanks. As far as we know, only the state-of-the-art 700-bar hydrogen tank designed by Toyota holds a hydrogen density of approximately 5.7 wt% H_2_ [12], just satisfying the present target of DOE. Ammonia (NH_3_) is also highly valued as a potential hydrogen storage option except compressed H_2_ gas, owing to its high hydrogen density (17.8 wt% and 0.120 kg H_2_ L^−1^ for gravimetric and volumetric H_2_ density), low storage pressure and stability for long-term storage as well as high flexibility in its utilization [13]. In this regard, NH_3_ can fulfill the demand to store the energy in time (stationary energy storage) and in space (energy export and import). However, NH_3_ encounters high energy demand in both synthesis and decomposition for indirect utilization by the release of H_2_. In case of liquid H_2_, in spite of a much higher volumetric density (0.071 kg H_2_ L^−1^) that even surpasses the ultimate targets of DOE at the temperature as low as -253 °C, the inevitable hydrogen loss resulted from heat transfer and a large amount of energy consumed to liquefy hydrogen severely impede its practical applications [8, 14]. As same as liquid H_2_, besides the much unavoidable energy consumption required in the high-pressurized compression, the high cost and latent safety risks of hydrogen refueling stations are the obstacles for the large-scale utilization in civilian vehicles. Admittedly, solid hydrogen storage materials [15] are the most acceptable hydrogen carriers and have received a great deal of attentions due to their ideal hydrogen density, reliable safety and numerous modification methods that have been developed to tailor their practical dehydrogenation capacities in recent years. Here, a comparison of some typical hydrogen mediums in terms of cost, hydrogen storage capacity and safety is summarized, as shown in Table 2.

**Table 1:** Current states vs targets for onboard H_2_ storage for light-duty fuel cell vehicles [11]

Storage targets	Gravimetric kWh kg^−1^ (kg H_2_/kg system)	Volumetric kWh L^−1^ (kg H_2_/L system)	Cost^1^ $/kWh ($/kg H_2_)
2020	1.5 (0.045)	1.0 (0.030)	$10 ($333)
2025	1.8 (0.055)	1.3 (0.040)	$9 ($300)
Ultimate	2.2 (0.065)	1.7 (0.050)	$8 ($266)
*Current status*^2^			
700 bar compressed (5.6 kg H_2_, type IV, single tank)	1.4 (0.042)	0.8 (0.024)	$15 ($500)

^1^Projected at 500,000 units/year

^2^FCTO Data Record #15,013, 11/25/2015: https://www.hydrogen.energy.gov/pdfs/15013 onboard storage performance cost.pdf

**Table 2:** A comparison between some typical lightweight materials and hydrogen mediums [16, 17]

Parameters	Solid				Non-solid	
Category	Metal hydride	Complex hydride	Microporous adsorbents		Liquid hydrogen	ammonia
Compound	MgH_2_	NaBH_4_	Activated carbon	MOF	H_2_	NH_3_
Gravimetric capacity (wt%)	7.6	10.7	2.1–2.6	6.1	1.4	1.89
Volumetric capacity (g L^−1^)	110	116	20	20	125	114
Cost	Low	High	Low	Low	Low	Low
Thermolytic kinetics	Slow	Slow	Fast	Fast	Fast	Slow
H_2_ release temperature (°C)	Very high (> 300)	Very high (> 500)	Low (-196 ~ 25)	Cryogenic (-196)	-253	350–900
Abundant	High availability	Schlesinger or Bayer method	High availability	High availability	High availability	High availability
Safety	Benign	Benign	Benign	Benign	Benign	Toxic and corrosive

In the mid and late of 2000s, the heavy intermetallic binary compounds were initially emerged as hydrogen storage materials owing to their good cycling performance and rapid kinetics under moderate conditions. However, the AB_2_ and AB_5_ types (ZrFe_2_, LaNi_5_, etc.), representative members of heavy metal alloys family, merely enable ≤ 2 wt% of hydrogen sorption because of the heavyweight and hydrogen non-absorptive trait of B side elements ^9, 18–19^. To meet the hydrogen storage targets given by DOE, scientists and researchers have been focusing toward novel lightweight hydrides [20–22]. Among these hydrogen materials, the most fascinating hydrides are magnesium-based materials (MgH_2_ as the host material) [23–25] and B-N compounds (borohydrides or ammonia borane) [26]. The gravimetric hydrogen densities of 7.6 wt% for MgH_2_ and 18.5 wt% for LiBH_4_ even exceed the value for on-board applications set by DOE. Recently, Shui’s group [27] synthesized a multilayered Ti_2_CT_x_ (T is a functional group) stack by incomplete hydrofluoric acid (HF) etching, and the as-prepared Ti_2_CT_x_ showed an unprecedented hydrogen uptake of 8.8 wt% H_2_ at room temperature and 60 bar H_2_, which is much higher than the ultimate targets of DOE. Unfortunately, most of light metal-based materials are considered to be irreversible under mild conditions, so a serious of tailoring strategies have been developed for hydrolysis and thermolysis. For example, it was found that ZrCl_4_ is an effective catalyst to considerably reduce the dehydrogenation temperature and activation energy for LiBH_4_ [28]. Furthermore, the hydrogen produced by the thermal decomposition is always accompanied with the emission of other explosive or toxic gas such as CO and/or B_2_H_6_ [29]. Generally, PEMFCs are very sensitive to the impurity of hydrogen, and even a little amount of impurity may cause the poisoning the catalysts [30]. Compared with the above approach, pure hydrogen supply from hydrolysis of light metal-based materials, including metal hydrides and borohydrides via reacting with water without external heat input, has a number of advantages, such as suitable operation temperature and well-controlled hydrogen release. Especially, hydrogen supply via hydrolysis is a self-humidification process, and such humid hydrogen can be conveyed directly into PEMFCs without dehumidification treatment and any performance loss [31]. Different from liquid H_2_ or gas-state hydrogen carriers that need further development and construction in infrastructures, such as the NH_3_/H_2_ pipelines, H_2_/NH_3_ refueling stations and liquefaction devices, the storage and transportation of metal hydrides and borohydrides hold low potential risk and low capital investment because they are largely compatible with the current transport infrastructure [13]. For Mg-based and Al-based materials, they can be stored and transported in the form of bulks. Moreover, the formation of a coherent passive layer deposited on the surface of bulks may prevent further oxidation of hydrolysable materials. With respect to borohydrides, NaBH_4_, an example of the family of borohydrides, is a well-known hydrogen carrier due to its high hydrogen-storage capacity (10.8 wt%) [32, 33]. It is easily dissolved in alkaline aqueous solution for safe, stable and long periods of storage, leading to a highly convenient transportation. Therefore, the currently available storage and transportation facilities and their regulation can be well utilized to increase the readiness for the adoption of light metal-based materials.

Hydrolysis enables hydrogen extraction from liquid water. However, the performance of hydrolysis reaction is subject to the operation temperature. The hydrogen generation rate will be significantly reduced in a low-temperature climate and the hydrolysis process could even be directly frozen in subzero circumstances. Methanol has a very low freezing point (-97 ℃); thus, hydrogen supply from methanolysis is considered optimal for real-time hydrogen production in low-temperature climate or subzero areas. At mild conditions, the reversible hydrogen storage systems like the metal-based hydrides have the advantages of fast hydrogen injection and durability for repeated recycling, whereas the hydrogen storage properties are plagued by the sluggish de-/hydrogenation kinetics, thermodynamic barriers (de-/rehydrogeneration temperature < 100 ℃, pressure < 10 atm) and cyclic performance [34]. In contrast, the device for hydrolysis hydrogen supply is very compact [35], and the hydrogen derived from water or light metal-based materials can be directly connected to the fuel cell to drive the motor. Significantly, water freight is safer and more convenient compared to high-pressure hydrogen storage and transportation. However, the controllability and utilization of enormous exothermicity of hydrolysis require further investigations.

In this review, we summarize the recent progress in the development of hydrolysis and alcoholysis of light metal-based materials, especially the Mg-/Al-based materials and borohydrides. To overcome the sluggish hydrolysis and low conversion, various methods have been developed, such as ball milling, catalysis, alloying, and solution modification. The different hydrolysis mechanisms of Al/Mg-based materials and sodium borohydride are discussed in detail. Furthermore, the recent advances in NaBH_4_ regeneration process from hydrolysis by-product are discussed. NaBH_4_ is considered as the most potential hydrolysable material.

## Hydrogen Generation from Hydrolysis or Alcoholysis

The typical hydrolytic materials include metals/hydrides, ammonia borane (NH_3_BH_3_, denoted as AB) and borohydrides. Hydrogen supply from NaBH_4_ hydrolysis was the most widely studied and has numerous advantages over the other hydrolytic materials, including half of hydrogen production from water, low operation temperature, environmentally benign by-product, well-controlled and high-purity hydrogen release [36–38], making it promising for on-board or onsite hydrogen supply. On the other hand, Mg- or Al-based materials are also widely discussed as hydrogen carriers, and they can supply high-purity H_2_ according to real-time demands via contacting with water. Compared to costly borohydrides, hydrogen supply from the light-metal materials is affordable and sustainable because of the abundant content in the earth crust and the mature recycling process in the industry. The following sections mainly emphasize the hydrolysis/alcoholysis of borohydrides, Mg-/Al-based alloys and hydrides.

### Highly Efficient Catalytic and Non-catalytic Alcoholysis/Hydrolysis of Borohydrides

Extensive efforts have been devoted to exploring highly efficient hydrolysis of borohydrides (NaBH_4_, Mg(BH_4_)_2_, LiBH_4_, etc.) or AB due to their excellent hydrogen storage capacities ^39–41^. For hydrogen application in fuel cells, if the water produced in the fuel cell part is redirected to LiBH_4_, then the H_2_ generation capacity may increase to 37.0 wt% [42]. Compared with the expensive LiBH_4_, NaBH_4_ with a 21.1 wt% H_2_ generation capacity (the water produced in the fuel cell part is recycled to react with NaBH_4_ and it is not taken into account in the case) is preferred as a more superior hydrolysable material, but its hydrolysis suffers from sluggish kinetics in neutral aqueous solutions. To lower the high kinetic barrier to an extent that would give a hydrogen generation rate closing to the requirement of practical applications, a variety of non-noble metal catalysts have been developed, such as Fe, Co, Ni or Pt, Ru, and Pd [43–47]. Especially, in the hydrolysis of borohydride aided by M_3_B (M = Cu, Ni, Fe), the catalytic activities are in the order of Cu < Ni < Co [48]. The Co-B-based types [49–52] are commonly admitted as reactive as noble metals and much more cost-effective, which exhibit saltant performance improvements. The enhanced performance results from the Co-B catalysts loaded on supports with a high surface distribution, where transition metals (Co, Ni, and Fe) act as active sites. The real hydrolysis by-product of NaBH_4_ is NaBO_2_·xH_2_O, and the real-time hydrolysis reaction is given as follows [53]:

1 $${\text{NaBH}}_{4} + \left( {2 + x} \right){\text{H}}_{2} {\text{O}} \to {\text{NaBO}}_{2} \cdot x{\text{H}}_{2} {\text{O}} + 4{\text{H}}_{2}$$

**Fig. 1:**
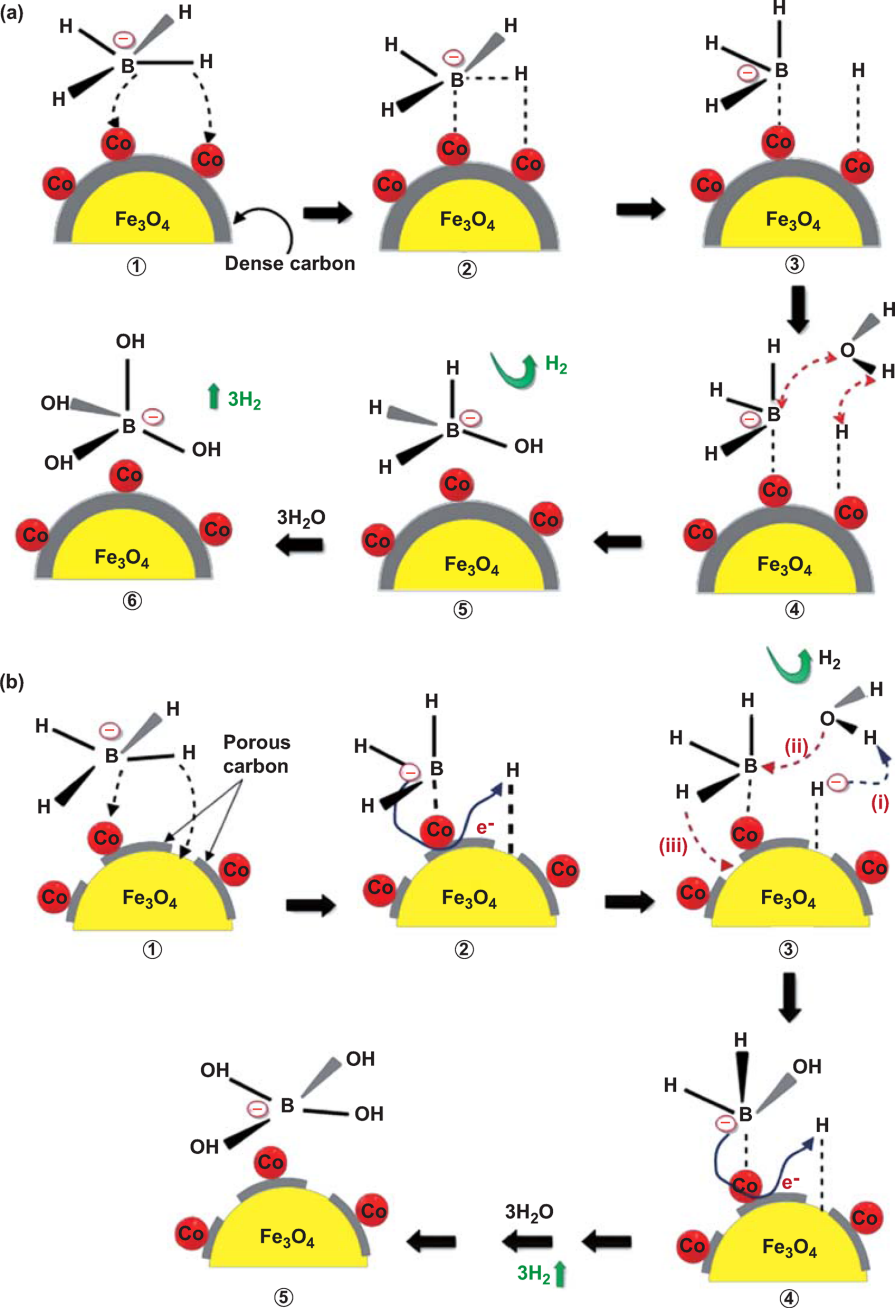
Schematic illustration for NaBH_4_ hydrolysis on **a** Fe_3_O_4_@C–Co and, **b** Fe_3_O_4_@C–X–Co (X = temperature). Reprinted with permission from Ref. [54]. Copyright 2019 Elsevier

**Fig. 2:**
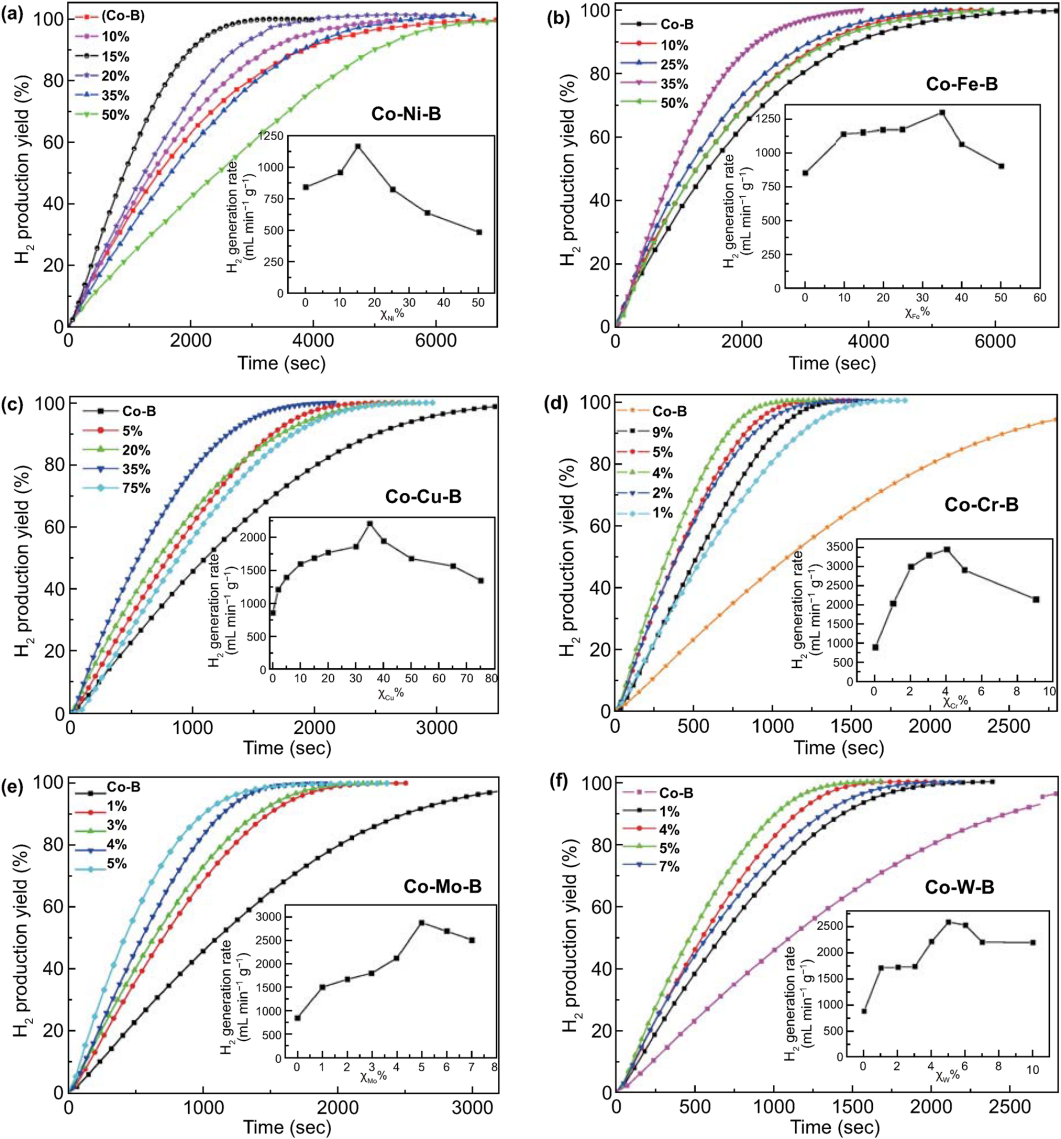
Hydrogen generation yield as a function of reaction time obtained by hydrolysis of alkaline NaBH_4_ (0.025 M) with **a** Co–Ni–B, **b** Co–Fe–B, **c** Co–Cu–B, **d** Co–Cr–B, **e** Co–Mo–B, and **f** Co–W–B with different *χ*_M_ values (where M = Ni, Fe, Cu, Cr, Mo, and W). Insets show the maximum H_2_ generation rate (*R*_max_) as a function of *χ*_M_. Reprinted with permission from Ref. [56]. Copyright 2010 Elsevier

That is, NaBH_4_ could produce four equivalents of hydrogen through the hydrolysis process. Recently, Appiah-Ntiamoah et al. [54] synthesized a novel catalyst with a core–shell structure, where Co was loaded upon Fe_3_O_4_@C “active” support. The unique properties of the “active” Fe_3_O_4_@C promoted a synergistic catalytic reaction involving Co, Fe_3_O_4_, and C during NaBH_4_ hydrolysis as shown in Fig. 1, delivering a hydrogen generation rate up to 1746 mL (g min)^−1^. Holbrook [55] believed that the hydrolysis mechanism with transition catalyst could be classified into five steps as shown in Fig. 1a. Firstly, the chemisorption of BH_4_^−^ on the metal atom site produces M-BH_3_ and M-H (step 1–3). Then, an electron from M-BH_3_ is transferred to the M site and BH_3_ is discarded, so the electronegative M site attracts H^+^ in water to form a new M-H. And a consumption of the two M-H can release one H_2_ molecule, then the BH_3_ legacy and OH– will form BH_3_(OH)^−^ (step 4–5). Subsequently, the stable intermediate BH_3_(OH)^−^ successively provides three active hydrogens, which will attack three H_2_O to form BOH_4_^−^ finally and release 3 mol of H_2_ (step 5–6). However, Fe exposed in the pores and Co could also from Fe_3_O_4_@C–Co to catalyze hydrogen release according to the mechanism proposed by Pena-Alonso via a synergistic effect as shown in Fig. 1b where hydrogen is firstly produced in the 3^rd^ step, and the entire reaction path is shortened. Moreover, the reusability and stability of Fe_3_O_4_@C–Co composite were investigated via successive catalytic runs, and there was negligible loss in the amount of H_2_ generated after 5 runs. The Fe_3_O_4_@C–Co composite showed high recyclability performance in catalytic activity and structural integrity, signifying its real-life application prospects. Furthermore, Patel's team [56] doped with various transition metals in Co-B-based binary catalysts and explored the hydrolysis properties as shown in Fig. 2. The Co–B-based ternary or quaternary catalysts may display better catalytic activity than binary catalysts. Table 3 summarizes recent advances on Co-based catalysts and their catalytic performances for NaBH_4_ hydrolysis. More information and applications about hydrogen production from NaBH_4_ for fuel-cell systems could be referred from a recent review [57].

**Table 3:** Comparison of some Co-based catalysts and their catalytic performance for NaBH_4_ hydrolysis

Catalyst	HGR (mL H_2_ (g_cat_ min) ^−1^)	Preparation method	Activation energy (kJ mol^−1^)	Refs.
Fe_3_O_4_@C–Co	1746	Hydrothermal method–thermal treatment	47.3	[54]
Co–Fe_3_O_4_–CNT	1213	Stepwise precipitation–microwave-assisted reduction	42.8	[58]
PAN/CoCl_2_–CNT nanofibers	1255	Electrospinning	52.9	[59]
Co/Fe_3_O_4_@C	1403	Wetness impregnation–chemical reduction	49.2	[44]
Co_3_O_4_ macrocubes	1498	Hydrothermal method	48.0	[60]
Co_3_O_4_ NA/Ti	1940	Hydrothermal treatment–annealing	59.8	[61]
Co–B/AT	1420	Impregnation–chemical reduction method	56.32	[49]
CoB/o–CNTs	3041	Wetness impregnation–chemical reduction	37.6	[50]
Co–La–Zr–B nanoparticle	102	Chemical reduction	51.00	[62]
p(AAm)–Co	1926	photopolymerization technique	39.7	[63]
NiCo_2_O_4_ hollow sphere	1000	Hydrothermal method	52.2	[64]
Carbon black supported Co–B	8034	Reduction–precipitation route	56.7	[65]
LiCoO_2_/Ru	3000	Microwave-assisted polyol process	70.4	[45]
Co–Ni–B/Cu sheet	14,778	Electroless plating	42.8	[51]
Co–W–P/Cu sheet	5000	Electrodeposition	22.8	[66]
Ru–SZ	9100	Sol–gel method	76	[46]
Co–Ni–Mo–P/γ-Al_2_O_3_	10,125	Electroless deposition	52.4	[67]
Co–B/TiO_2_	12,503	Chemical reduction	51.6	[52]
Co–P/Cu sheet	2275.1	Electroless plating	27.9	[68]
CoeP/Cu sheet	3300	Electroless plating	60.2	[69]
Co–P/Cu sheet	5956	Electroplating	23.9	[70]
Flower-like Co–P	1647.9	Electroless plating	47.0	[71]
Co–Mo–Pd–B	6023	Chemical reduction	36.4	[72]

**Fig. 3:**
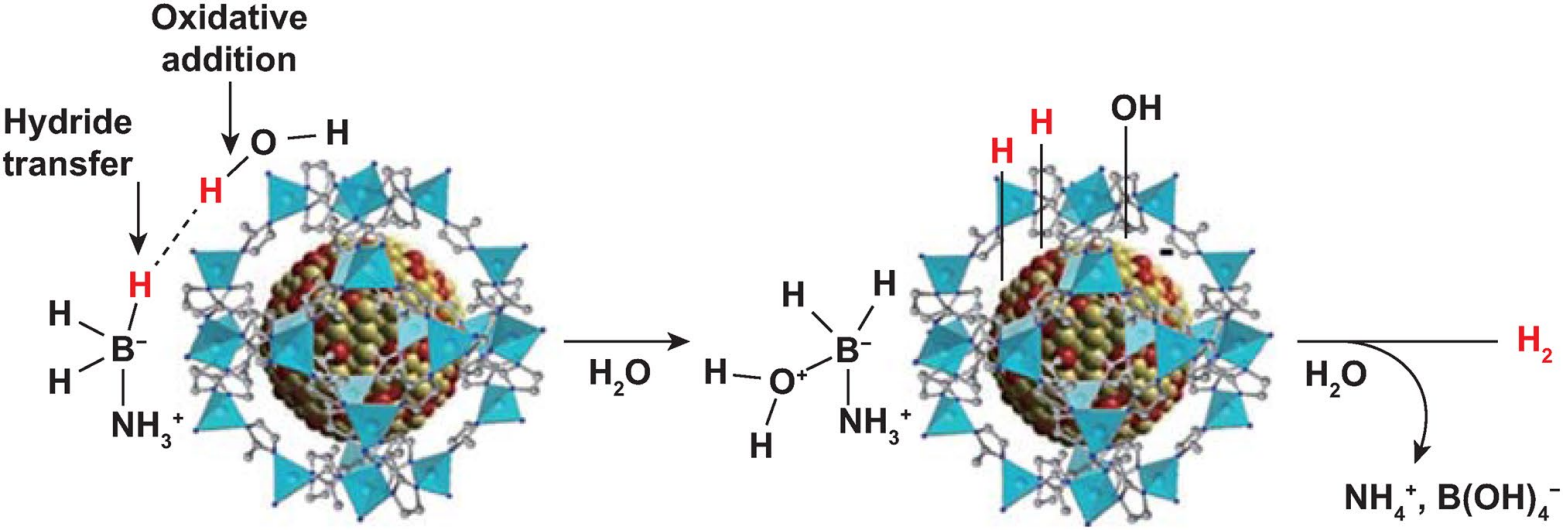
Proposed mechanism for the hydrolysis of AB catalyzed by NiPt@ZIF-8. Reprinted with permission from Ref. [77]. Copyright 2018 American Chemical Society

AB is considered as a leading contender in promising chemical hydrogen-storage materials for various applications due to its high hydrogen density (19.6 wt%) and high stability both in solid state and solution under ambient conditions, as well nontoxicity and high solubility [33, 73]. It can release three equivalents of hydrogen vis thermolysis, but the third-step dehydrogenation requires more than 1200 ℃. Similarly, the developed catalysts for the hydrolysis of NaBH_4_, such as noble metal-based NPs and Co-based NPs deposited on supports, can also impel AB hydrolysis as well. Li et al. [74] synthesized CVD-Ni/ZIF-8 by chemical vapor deposition, which could promote ammonia borane to release 3 equivalents of hydrogen in 13 min. Later, Wang et al. [75] deposited Ni NPs in ZIP-8 by NaBH_4_ reduction method, which promoted AB to complete reaction in 0.3 M NaOH solution within 5 min with a TOF value of 85.7 mol_H2_ mol_cat_^−1^ min^−1^. Interestingly, it was found that H^+^ in the acid could slow the reaction, and a certain concentration of OH^−^ remarkably improved hydrogen evolution. Therefore, a switch was designed to control hydrogen supply by adjusting the pH value of the solution. In addition, the reusability of the nanocatalyst NiNPs/ZiF-8 was examined by the continuous addition of a new proportion of AB aqueous solution when the previous run was completed. It was found that the activity of NiNPs/ZiF-8 was essentially retained until the fifth run and there was almost no loss in the amount of H_2_ generated during the cycling test. He et al. [76] also got the same result that OH^−^ in aqueous solution is crucial in determining the hydrolysis kinetics of AB through the kinetic isotope effect (KIE). Wang et al. [77] further explored the hydrolysis mechanism of Ni_2_Pt@ZIF-8 and found that OH^−^ acted as a catalyst promoter, making the NP more electron-rich, which could favor the oxidative addition of water, as shown in Fig. 3. The presence of OH^−^ boosts H_2_ evolution that becomes 87 times faster than in its absence with Ni_2_Pt@ZiF-8. The kinetic isotope effects using D_2_O showed that cleavage by oxidative addition of an O–H bond of water onto the catalyst surface is the rate-determining step of this reaction, enabling significant progress in catalyst design toward convenient H_2_ generation from hydrogen-rich substrates in the near future.

**Fig. 4:**
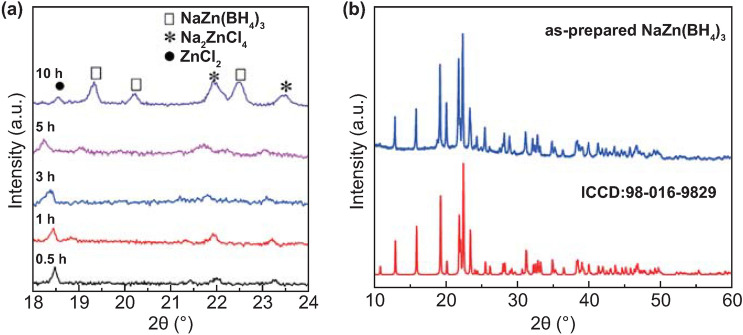
XRD patterns of **a** NaBH_4_-ZnCl_2_ composites ball-milled for different durations. Reprinted with permission from Ref. [79], Copyright 2017 Elsevier, and **b** purified NaZn(BH_4_)_3_ and its standard PDF card. Reprinted with permission from Ref. [36], Copyright 2017 Royal Society of Chemistry

Although the introduction of the catalyst can enhance the reaction to some extent, the difficulty and cost in recovering the catalyst, however, is an issue. Therefore, it is required to develop catalyst-free hydrogen supply systems from light-metal-based materials. Recently, Ouyang and co-workers investigated the non-catalytic hydrolysis of some borohydrides [36, 78, 79]. For instance, they found that the hydrogen generation rate for NaBH_4_ hydrolysis could be accelerated by doping with ZnCl_2_ without involving catalysts. It was found that NaBH_4_-35 wt% ZnCl_2_ achieved the optimal hydrogen yield of 1964 mL g^−1^ H_2_ with a considerable hydrogen production rate of 1124 mL g^−1^ within only 5 min [79]. Interestingly, they observed the existence of NaZn(BH_4_)_3_ (Fig. 4) after ball milling the mixture of NaBH_4_-ZnCl_2_ and further investigated the hydrolysis performance of pure NaZn(BH_4_)_3_ [36]. The results showed that NaZn(BH_4_)_3_ enabled the hydrogen release of 1740 mL g^−1^ in 5 min with a total hydrogen yield up to 97%. Because the ligands neighboring the metal cations in the borohydride involve the hydrogen elimination barrier and the stability of BH_4_^−^ [80], they introduced NH_3_ to achieve a rate-controlled hydrogen supply of NaZn(BH_4_)_3_ by forming its ammoniate. Similarly, they also studied the effect of ammonia complex number on hydrogen production kinetics by Mg(BH_4_)_2_ hydrolysis [78]. Obviously, the hydrogen evolution behaviors could be well-controlled via altering ammonia complex number upon Mg(BH_4_)_2_, whereas it sacrificed hydrogen yield. The hydrogen yields of Mg(BH_4_)_2_·0.5NH_3_, Mg(BH_4_)_2_·NH_3_, Mg(BH_4_)_2_·2NH_3_, Mg(BH_4_)_2_·3NH_3_, and Mg(BH_4_)_2_·6NH_3_ are 2376, 2029, 1780, 1665, and 1180 mL (H_2_) g^−1^, respectively. Similarly, Mg(BH_4_)_2_ can possess different hydrolytic behaviors when coordinated with various organic ligands (including Mg(BH_4_)_2_ × xE_2_O, Mg(BH_4_)_2_ × diglyme and MgBH_4_ × 3THF), with the larger the ligand and the higher the denticity, and the smaller amount of B_2_H_6_ being produced [81].

As is well known, the hydrogen generation performance would deteriorate markedly followed by temperature decrease. To solve this issue, alcoholysis and alcoholysis/hydrolysis composite hydrogen generation systems for NaBH_4_ have been developed [37, 82–85]. For example, hydrogen release from NaBH_4_ in ethylene glycol/water solutions in the presence of CoCl_2_ catalyst could be quickly launched even at -10 ~ 20 °C, fulfilling 100% of fuel conversion within only a few minutes. What’s more, the hydrogen density of the alcoholysis/hydrolysis composite system with optimized composition may reach 4 wt%. This demonstrated that a superior-performance hydrogen generation system with a wide range of operational temperature may be developed for practical hydrogen source for mobile/portable applications [37].

For LiBH_4_ hydrolysis, the catalyst-free hydrolysis reaction never surpasses 50% of its theoretical yield due to the low solubility of the LiBO_2_-based by-product in water that deposits on LiBH_4_ and limits the full utilization of the hydride [86]. Kojima et al. [87] reported that the hydrogen densities increased with the increase in the dropped water (H_2_O/LiBH_4_) and followed by a reduction. These densities may show maximum values at H_2_O/LiBH_4_ = 1.3. To enhance the sluggish kinetics and low conversion efficiency for LiBH_4_ hydrolysis, a series of strategies have been adopted toward H_2_ release at approximately a stoichiometric equivalent, including the hydrolysis system of LiBH_4_ doped with multiwalled carbon nanotubes (MWCNTs) [88] or diethyl ether addition [89], the non-catalytic hydrolysis of LiBH_4_/NH_3_BH_3_ composite system [90], and the catalytic hydrolysis reaction system of LiBH_4_ solution over nano-sized platinum dispersed on LiCoO_2_ (Pt–LiCoO_2_) [91], etc. Considering the affordability and sustainability, it is imperative to develop low-cost and non-noble metal catalysts that hold similar activity and stability with noble metals in the conversion and utilization of LiBH_4_ hydrolysis system. Recently, Zhu’s group [92] firstly adopted the transition-metal chlorides (CoCl_2_, NiCl_2_, FeCl_3_) to promote the hydrolysis behaviors of LiBH_4_. Among the above catalysts, CoCl_2_ showed faster hydrogen kinetics, delivering a hydrogen generation rate ranging from 421 to 41,701 mL H_2_ min^−1^ g^−1^ with a maximum conversion of 95.3%. These values are much higher than the value of 225 mL H_2_ min^−1^ g^−1^ with Pt-LiCoO_2_. Moreover, NH_3_ was introduced to tailor the uncontrollable kinetics of LiBH_4_ by forming its ammoniates (LiBH_4_·xNH_3_, x = 1, 2, 3). In the presence of CoCl_2_, LiBH_4_·xNH_3_ could stably release over 4300 mL H_2_ g_LiBH4_^−1^ with a hydrogen capacity of ~ 7.1 wt% and a H_2_ yield of 97.0%, while it reacts with a stoichiometric amount of H_2_O. However, the difficulty in regenerating the utilized LiBH_4_ and the associated high cost hamper their large-scale applications. In the near future, developing convenient and economical methods for LiBH_4_ regeneration is a linchpin, as it acts as hydrogen carrier in off-/on-board applications.

### Hydrogen Production via Hydrolysis of Mg-based Alloys or Its Hydrides

**Fig. 5:**
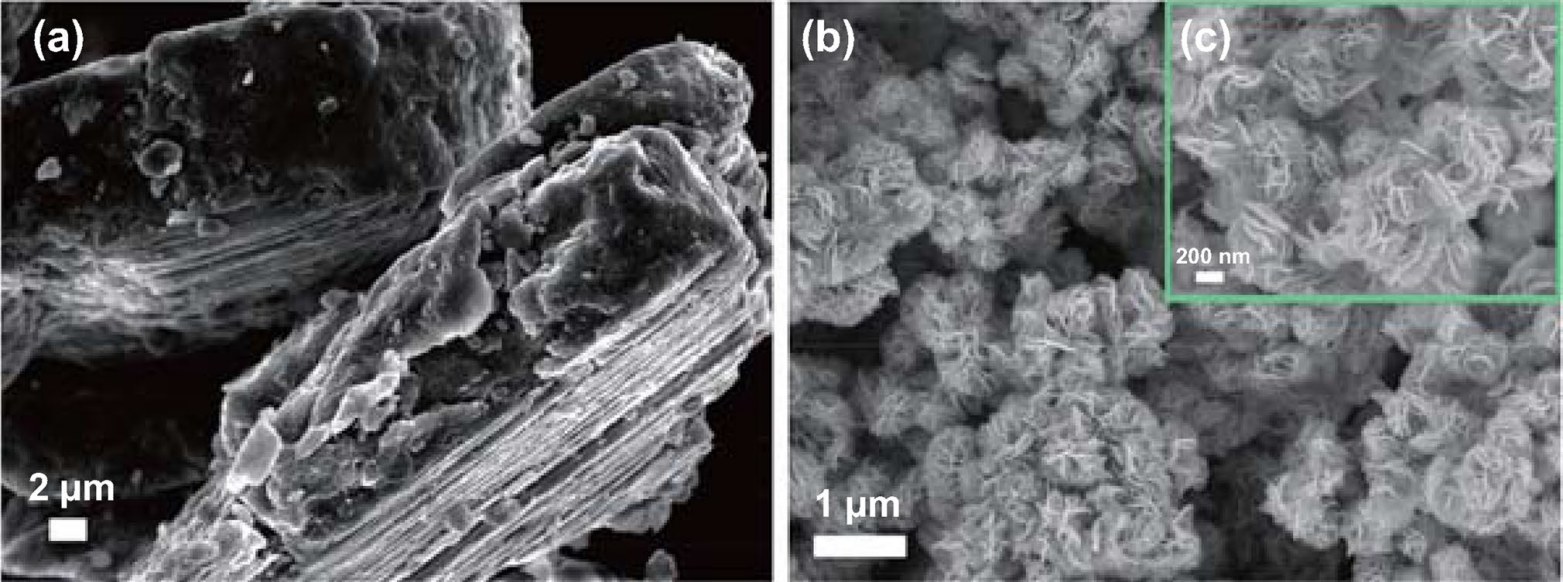
SEM images of **a** bulk and **b** as-prepared MoS_2_, **c** high-magnification SEM image showing a small zone of the as-prepared MoS_2_. Reprinted with permission from Ref. [100]. Copyright 2017 Elsevier

**Fig. 6:**
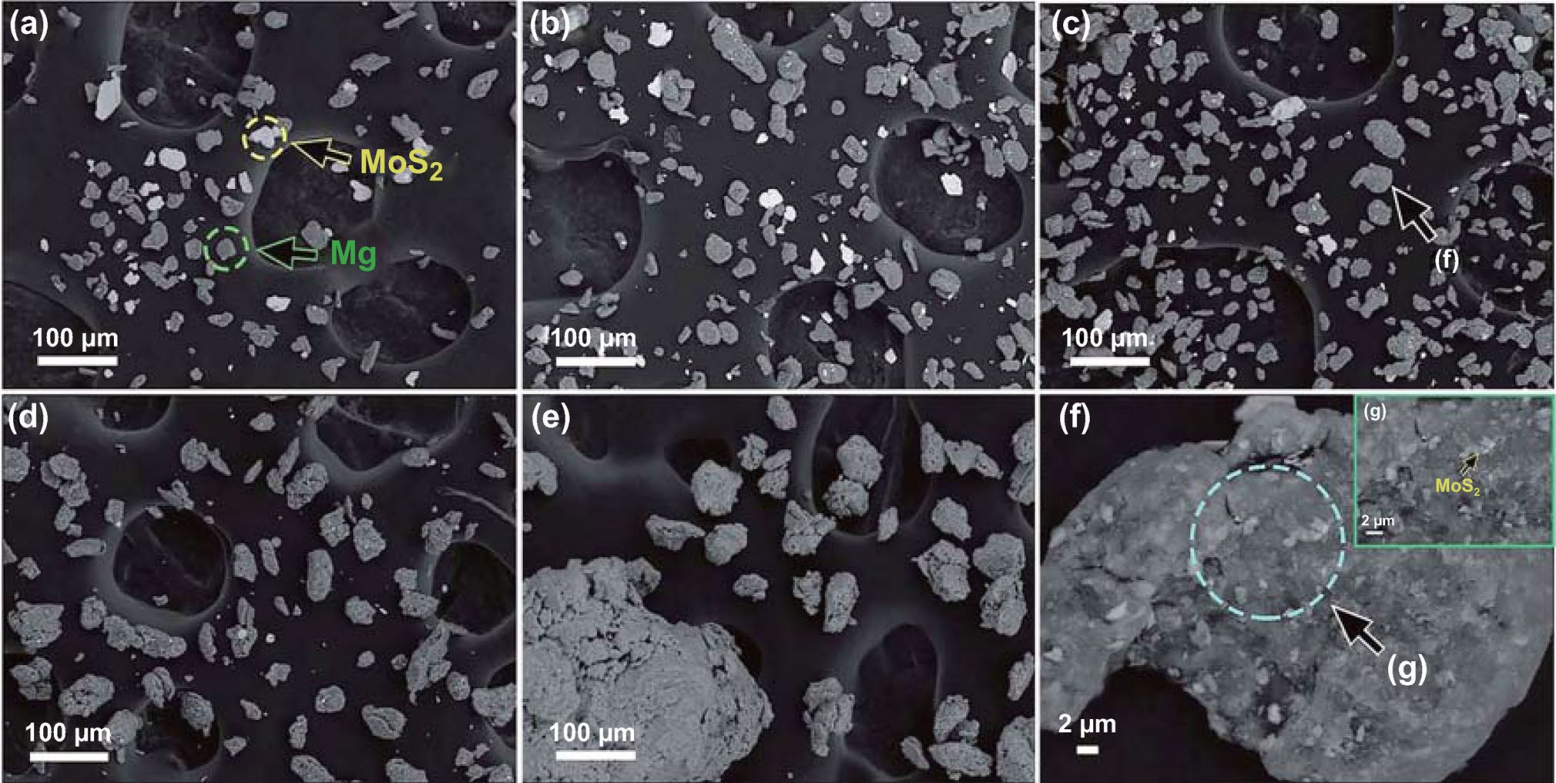
SEM images of the Mg-10 wt% MoS_2_ composite milled for various durations: **a** 0.1 h, **b** 0.5 h, **c** 1 h, **d** 3 h, and **e** 5 h, **f** and **g** high-magnification SEM images showing a small zone of the Mg-10 wt% MoS_2_ composite milled for 1 h. Reprinted with permission from Ref. [99]. Copyright 2017 Royal Society of Chemistry

**Fig. 7:**
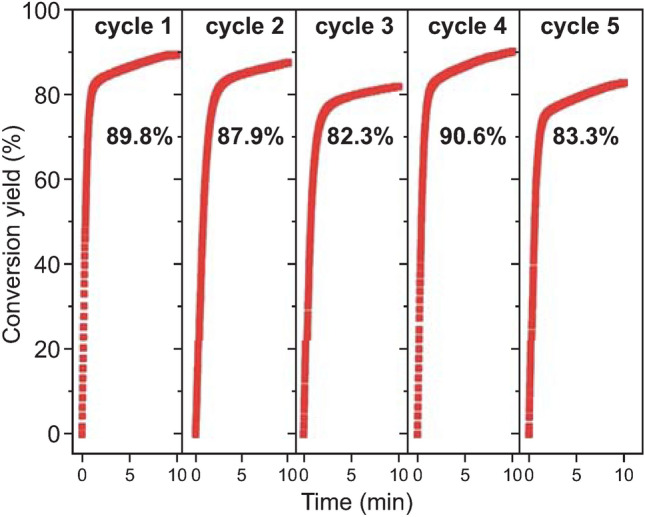
Cyclic curve of hydrogen evolution via hydrolysis of Mg-10 wt% retrieved MoS_2_ milled for 1 h in seawater. Reprinted with permission from Ref. [99]. Copyright 2017 Royal Society of Chemistry

Compared to borohydrides, the hydrolysis from light metals and metal hydrides for down-to-earth hydrogen supply has a number of advantages, including low-cost, abundant element contents, environmentally benign products of oxidation, etc. [38, 93–95]. Generally, it is widely accepted that the hydrolysis reaction of Mg or MgH_2_ is rapidly interrupted by a passive Mg(OH)_2_ layer deposited on the surface of Mg-based materials, leading to poor hydrolysis performance. To date, numerous methods, such as ball milling, alloying, aqueous solution modification or catalysis [96–99], have been applied to enhance the sluggish kinetics. Recently, Ouyang’ group [100] synthesized flower-like MoS_2_ spheres via a one-step hydrothermal method. The as-prepared MoS_2_ composes of many uniform spherical nanoparticles (Fig. 5), resulting in larger surface areas than its bulk counterpart. The Mg-10 wt% MoS_2_ composite could release over 90% of theoretical hydrogen capacity in 1 min. Also, they investigated the catalytic effects of the transition metal Mo and its compounds (MoS_2_, MoO_2_, and MoO_3_) upon hydrolysis of Mg in seawater [99]. The results showed that the distribution of MoS_2_ catalyst in the Mg matrix became increasingly homogeneous with the increase in milling time (Fig. 6). The unique structure and uniformly dispersed MoS_2_ could significantly accelerate the hydrolysis process of Mg. Moreover, the reusability and stability of MoS_2_ were investigated via successive catalytic runs. As shown in Fig. 7, there was a slight drop in the amount of H_2_ generated after 5 runs, and the catalytic activity of retrieved MoS_2_ was completely retained without decrease in H_2_ evolution rate. They believed that the markedly enhanced activity could be attributed to the synergistic effect of grinding and the galvanic corrosion between Mg- and Mo-based additives.

In addition to doping catalysts, alloying and ball milling have been proved to be effective means to enhance the hydrolysis performance of Mg. Ouyang et al. [97, 102–106] systematically studied the hydrolysis behaviors of Mg-RE alloy and its hydrides. They found that rare-earth elements could facilitate the hydrogen absorption of Mg-based alloys, resulting in higher hydrogen yields for the hydrolysis of hydrogenated Mg-RE. Ma et al. [107] revealed that Ni could promote the hydrogenation of CaMg_1.9_Ni_0.1_ under room temperature, as opposed to 450 °C for pure CaMg_2_. Thus, the H-CaMg_1.9_Ni_0.1_ could achieve a hydrogen yield of 1053 mL g^−1^ in only 12 min, approximately twice as much as that of CaMg_1.9_Ni_0.1_. In this regard, they doped a small amount of Ni toward CaMg_2_ via ball milling [108]. The hydrogen yield of the hydrogenated CaMg_2_-0.1Ni sample could increase from 853 to 1147 mL H_2_ g^−1^ in 5 min with hydrogenation durations ranging from 0.5 to 1.5 h. On the other hand, Ouyang et al. [109] found that the hydrolysis properties of Mg can be greatly enhanced with the addition of expanded graphite by plasma-assisted milling. The obtained Mg-graphite composite could release 614.3 mL H_2_ g^−1^ in 25 min with a hydrolysis conversion rate of 83.5%. They also synthesized refined hydrogenated MgLi (H-MgLi) by reactive ball milling [110], producing ~ 15.8 wt% hydrogen in 5 min. As same as NaBH_4_, the hydrogen generation behaviors of Mg would deteriorate markedly followed by decreased temperature. To remove the troublesome freezing issue of the water solution system in low-temperature conditions, Ouyang et al. [111] adopted pure methanol, methanol/water and methanol/ethanol solutions to react with CaMg_2_ alloy and its hydrides for hydrogen generation. The as-prepared CaMg_2_ could generate 858 mL H_2_ g^−1^ within only 3 min at room temperature, while it reacted vigorously with methanol, as opposed to a low hydrogen yield with ethanol and water (395 and 224 mL H_2_ g^−1^ within 180 min, respectively). Even at − 20 °C, there was still over 600 mL H_2_ g^−1^ released at a conversion rate of 70.7% within 100 min for methanolysis, demonstrating its prominent advantage for hydrogen production, especially in winter or subzero areas.

**Fig. 8:**
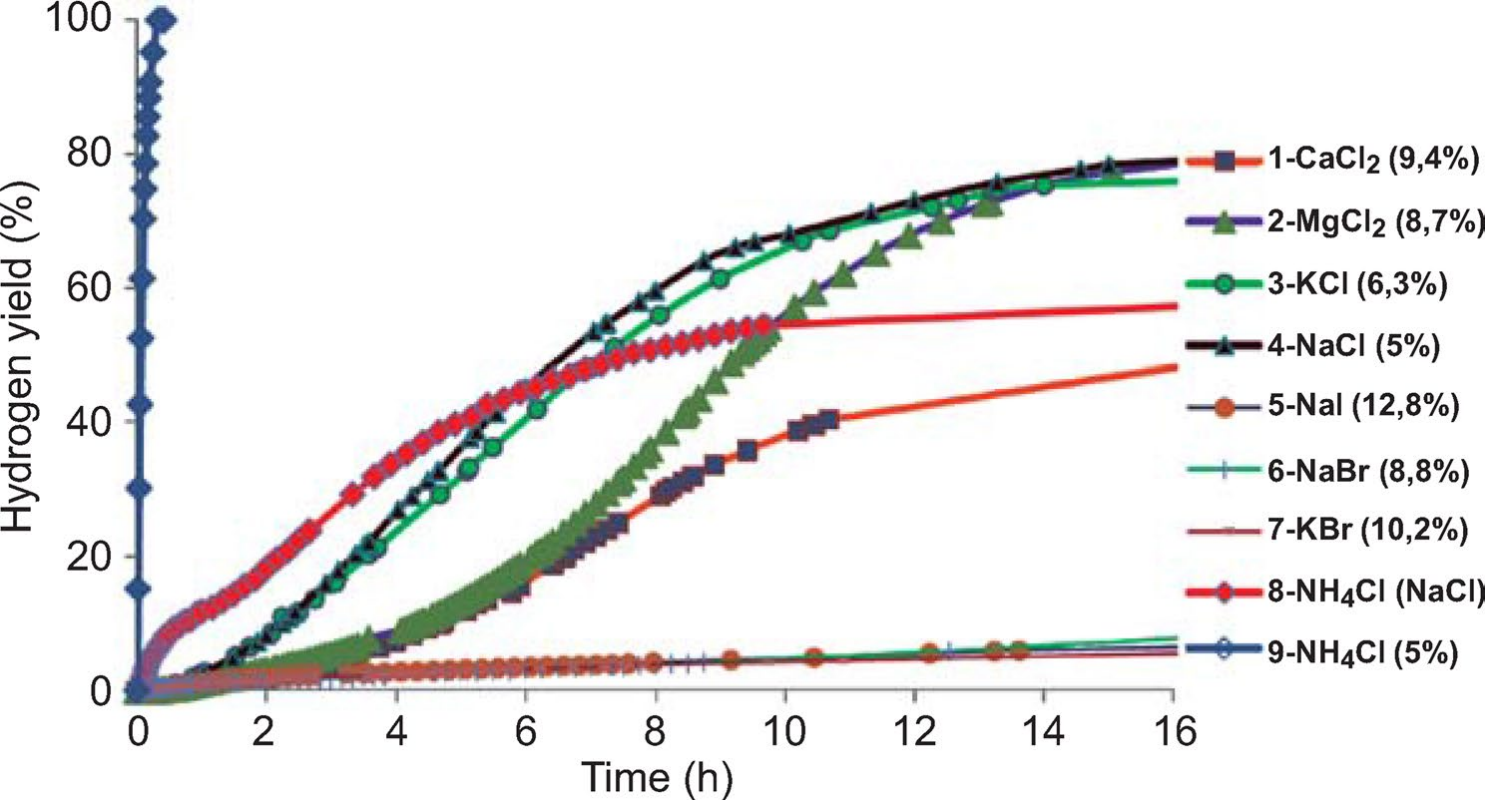
Yields of hydrogen release due to magnesium powder oxidation in the presence of alkali, alkaline earth and ammonia halides (1 g of Mg in 30 mL of salt solution). Halide concentration was maintained approximately the same: 0.85 M (curves 1–7); 0.93 M for NH_4_Cl (curve 9) and 0.31 M NH_4_Cl + 0.85 M NaCl (curve 8). Reprinted with permission from Ref. [101]. Copyright 2014 Elsevier

Aqueous solution modification is also an effective strategy to tailor the hydrogen behaviors of Mg-based materials. In real application, large excess of water is required to ensure complete hydrolysis of Mg, resulting in significant capacity loss. The formation of insoluble Mg(OH)_2_ enables simple separation and repeated using of water, which minimizes the hydrogen capacity loss caused by the excessive water. In this regard, Li et al. [112] solved the issue by using MgH_2_ nanoparticles together with the promotion effect of MgCl_2_ solution. A near-theoretical amount of H_2_ (1820 mL g^−1^) was released within 20 min in 1 M MgCl_2_ solution without any pretreatment of the MgH_2_ nanoparticles (800 nm). By separating Mg(OH)_2_ through filtration and recycling the MgCl_2_ solution, the hydrogen capacity of this system may approach the theoretical value of 6.45 wt% with continuous MgH_2_ and water feeding. Recently, Tan et al. [113] reported that the hydrolysis performance of Mg_2_Si could be notably improved by using NH_4_F solution. The fluorine ion was introduced to restrain the release of silanes during the hydrolysis reaction of Mg_2_Si. Due to its high chemical affinity to silicon ion, it is possible for F^−^ to break the Si–H bond and form H_2_ and SiF_6_^2−^ in aqueous solution. As the concentration of the NH_4_F solution increased to 13.0%, the hydrogen yield of Mg_2_Si reached the maximum, producing 616 mL H_2_ g^−1^ in 30 min at 25 °C. The L.G. Sevastyanova et al. [101] systematically explored the effect of salt solutions and the transition metals on magnesium hydrolysis (Fig. 8) and found (1) the NH_4_Cl solution exhibited the fastest initial reaction rate, but the conversion yield reached the maximum in NaCl solution, (2) aqueous solutions of alkaline or alkali earth metal chlorides at a salt content over 3 wt% would effectively improve the hydrolysis performance (the optimal amount being 4–15 wt%), (3) the transition metals can also cause reduction of the hydrogen yield if it is over 10 wt%. Correspondingly, Table 4 lists the varieties of some Mg-based materials and their hydrolysis properties. Nearly all hydrolysis materials enable the solution concentration being at least 3 wt% and the amount of oxidation addition not exceeding 10 wt%.

**Table 4:** Comparisons of some ball-milling Mg-based materials and their hydrolysis performances

Materials	Solution	Hydrogen yield (%)	HGR (mL H_2_/(g min)^−1^)	Activation energy (kJ mol^−1^)	Refs.
Mg–10 wt% MoS_2_	3.5% NaCl solution	90.4% in 1 min	–	12.9	[100]
Mg–10 wt% MoO_3_	3.5% NaCl solution	91.7% in 10 min	2423	12.1	[99]
Mg–10 wt% MoO_2_	3.5% NaCl solution	88.0% in 10 min	1933	14.3	[99]
Mg–10 wt% Mo	3.5% NaCl solution	86.5% in 10 min	751	27.6	[99]
Mg–10 wt% CoCl_2_	Pure water	93.4% in 30 min	524	–	[114]
Mg–10 wt% FeCl_3_	Pure water	98% in 2 min	1479.7	–	[115]
H–Mg_3_La	Water	88% in 20 min	43.8	–	[105]
H–Mg_17_La_2_	Water	60.1% in 21 min	40.1	–	[105]
H–Mg_3_CeNi_0.1_	Pure water	57.4% in 10 min	276	–	[97]
H–CaMg_1.9_Ni_0.1_	Pure water	94.6% in 12 min	–	32.9	[107]
H–MgLi	Pure water	82% in 5 min	–	10.6	[110]
H–MgLi	1 M MgCl_2_ solution	90% in 30 min	–	24.6	[110]
Mg–10% In	Seawater at 30 °C	93% in 10 min	444	12.4	[116]
Mg–10% In	Methanol at 20 °C	95% in 1 min	6900	–	[116]
Mg–Mg_2_Cu eutectic alloy	3.5% NaCl solution	90% in 20 min	–	36.91	[117]
Mg–Mg_2_Sn eutectic alloy	3.5% NaCl solution	90% in 20 min	–	38.19	[117]
Mg–90wt% NdNiMg_15_	3.5% NaCl solution	100% in 15 min	60	–	[118]
Mg–Mg_2_Si	0.5 M MgCl_2_ solution	90% in 60 min	–	9.5	[93]
Mg–5 wt% G–5 wt% Ni	3.5% NaCl solution	95% in 2 min	–	14.34	[119]
Mg–10wt%Nd_2_O_5_	3.5% NaCl solution	100% in 30 min	–	31.46	[119]
30 wt% Ca-Mg hydrides	Deionized water	69.9% in 5 min	–	8.3	[120]
Mg-3% mol Al	Water	93.86% in 60 min	455.9	–	[121]
4MgH_2_-LiNH_2_	Water	72.7% in 50 min	887.2	–	[38]
MgH_2_	4.5 wt% NH_4_Cl solution	81% in 30 min	–	30.373	[98]
(Mg10Ni)_95_Ce_5_	Seawater	87% in 15 min	149.4	33.8	[122]
(Mg10Ni)_95_Ce_5_-EG-MoS_2_ composite	Seawater	95% in 1 min	773	14.5	[122]
(Mg–10Ni)_85_La_15_	Distilled water	12.8% in 135 min	0.4683	–	[123]
(Mg10Ni)_95_Ce_5_	3.5 wt% NaCl solution	92% in 200 min	–	27.11	[124]
Mg10Ni–5wt%EG–5wt%MoS_2_	3.5 wt% NaCl solution	91% in 5 min	148.16	9.26	[125]
Mg–25wt%Ni	3.5 wt% NaCl solution(48 °C)	60.3% in 30 min	48.28	9.57	[126]
Mg–30wt%Ce	3.5 wt% NaCl solution(48 °C)	85% in 30 min	171.88	14.65	[126]
Mg–30wt%La	3.5 wt% NaCl solution(48 °C)	90.2% in 30 min	74.52	23.88	[126]
Mg_10_Ni–5 wt% MoS_2_	3.5 wt% NaCl solution	67% in 15 min	1480	18.79	[127]

### Hydrogen Production via Hydrolysis of Al-based Alloys or Its Hydrides

The distribution of aluminum is more abundant than magnesium, being third only to oxygen and silicon. Aluminum is a safe and cheap metal as well as electrochemically active element; thus, it may be a more appropriate candidate for the process of hydrogen production [31, 128]. The catholic use of aluminum is for the applications in batteries [129], like the aluminum–air battery that has an aluminum-based anode. While this aluminum-based battery has potential prospect in electric vehicles, it is inhibited by the undesirable parasitic corrosion reaction or the formation of a dense oxide layer. But the reaction actually produces hydrogen.

In addition, OH^−^ can dissolve the passive layer and form AlO_2_^−^ to generate hydrogen even at room temperature. Taking the most commonly used NaOH solution as an example, the hydrogen generation is proposed as follows [130]:

2 $$2{\text{Al}} + \, 6{\text{H}}_{2} {\text{O}} + \, 2{\text{NaOH}} \to \, 2{\text{NaAl}}\left( {{\text{OH}}} \right)_{4} + 3{\text{H}}_{2}$$

3 $${\text{NaAl}}\left( {{\text{OH}}} \right)_{4} \to {\text{NaOH}} + {\text{Al}}\left( {{\text{OH}}} \right)_{3}$$

4 $$2{\text{Al}} + \, 6{\text{H}}_{2} {\text{O}} \to \, 2{\text{Al}}\left( {{\text{OH}}} \right)_{3} + 2{\text{H}}_{2}$$

5 $$2{\text{Al}} + \, 4{\text{H}}_{2} {\text{O}} \to 2{\text{AlOOH}} + 3{\text{H}}_{2}$$

Initially, the hydrogen generation reaction consumes sodium hydroxide, but when the NaAl(OH)_4_ concentration exceeds the saturation limit, it leads to the NaOH regeneration process accompanying aluminum hydroxide formation. Therefore, only water is consumed during the whole hydrogen supply as shown by the reactions (4 and 5), and the hydrolysis by-products are the non-polluting bayerite (Al(OH)_3_) and boehmite (AlOOH) [2, 131, 132]. Though the addition of OH^−^ is considered as the simplest and the most effective approach for promoting the Al/H_2_O reaction [133], the use of an aqueous NaOH solution causes corrosion of system apparatus. Therefore, novel technologies that enable a combination of a minimized quantity of NaOH and rapid H_2_ generation kinetics are highly desirable. Wang et al. [134, 135] found that a combined usage of sodium hydroxide (NaOH) and sodium stannate (Na_2_SnO_3_) can simultaneously address the Al/H_2_O reaction kinetics and alkali corrosion problems. The addition of a small amount of Na_2_SnO_3_ causes a remarkable decrease of NaOH concentration without compromising the hydrogen generation performance of the system. In comparison with the traditional Al/H_2_O system using aqueous NaOH solution, the new system exhibits a series of advantages in hydrogen generation performance, manipulability and adaptability; all are relevant to the development of practical aluminum-based hydrogen generation systems for mobile or portable applications. Notably, aluminum can be regenerated from the by-products by mature industrial technologies, the Bayer process [136] from bauxite ore (AlOOH) and the Hall–H′eroult process [137] from alumina.

Since Belitskus [130] first proposed the Al–water reaction to provide hydrogen in the 1970s, crucial efforts have been put into action to overcome the hydrolysis obstacle caused by the formation of the Al_2_O_3_ layer. Ball milling, as a frequently used method for increasing the hydrolysis performance of Mg-based materials, has proved to be effective for Al-based materials [138–142]. Yan et al. [140] milled an Al-10 mol% LiH-10 mol% KCl mixture for 10 h and obtained a hydrogen yield of 97.1% in 10 min at 60 ℃. The effects of metal chlorides to aluminum were similar to magnesium in hydrolysis. Firstly, chlorides can decrease the grain size during ball milling, and secondly, chlorides can also raise galvanic corrosion of magnesium or aluminum. Thirdly, Cl^−^ could damage the Mg(OH)_2_ or Al(OH)_3_ layer. Except mechanical activation by ball milling, torsional pressure and ultrasonic assistance, chemical activation of aluminum, such as by alloying, is also applicable. Originally, mercury was utilized for chemical activation of aluminum [143]. While mercury is a toxic substance and is not recommended for use in large scale, the new method of alloying to activate aluminum for aluminum–water reaction is sought after [144–147].

**Fig. 9:**
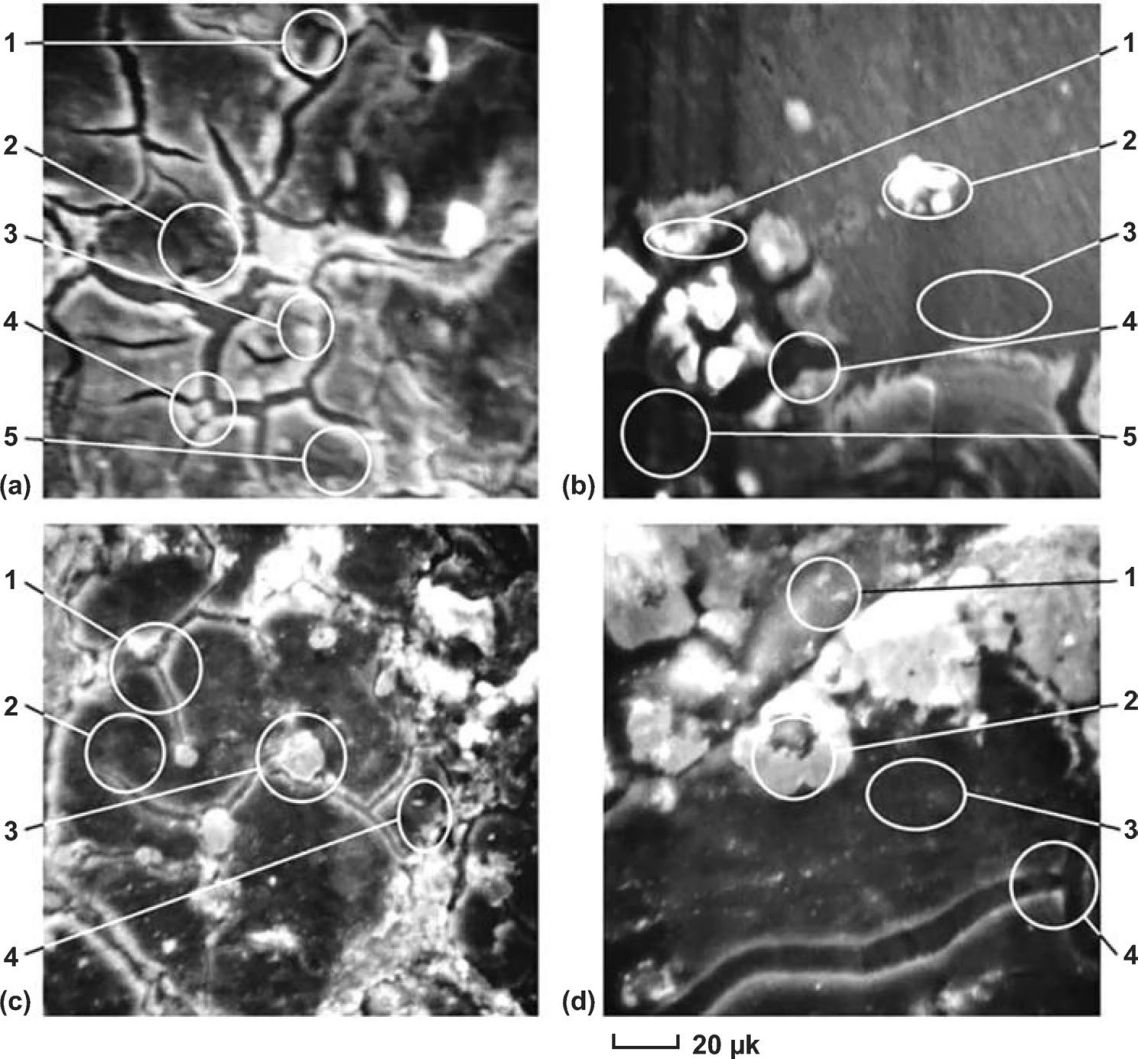
SEM images of multicomponent aluminum alloy (Ga:In:Sn:Zn:Al = 5*.*3:2.0:5.4:7.3:80.0) sections (× 800). **A** After preparation, **B** after annealing at 450 °C for 20 h. **C** and **D** after storing as-cast and annealed alloys for 1 month. Reprinted with permission from Ref. [144]. Copyright 2005 Elsevier

It has been confirmed that the hydrolysis properties have been enormously boosted up by alloying low melting point metals (LMPM) such as Ga, In, Sn and Zn with Al. Bulychev et al. [144] investigated the hydrolysis properties of aluminum alloy containing different accounts of LMPM. They found that the hydrogen supply virtually did not proceed without the presence of gallium, and the absence of indium in the alloy also led to a sharp decrease in the hydrolytic ability. But this alloy showed a terrible stability even stored under an inert atmosphere or in vacuum. They believed that this might be related to the presence of dispersed solid phases and a liquid phase (eutectic) distributed over the grain boundary space (Fig. 9**)**. Parmuzinaa [145] held a point of view that the liquid eutectics based on gallium brought about eutectic penetration into aluminum grain boundaries, which destructed the inter-crystal contacts and resulted in the formation of aluminum monocrystal powders covered by eutectic thin film. Dong et al. [148] demonstrated that the presence of a liquid phase in the Al–Ga and Al–Ga–In–Sn alloys was decisive for the alloys to react with water and produce H_2_ with an average yield of 83.8% in all 80 trials. The reaction temperature correlated well with the reported Al–Ga binary eutectic melting point of 26.6 ℃ and Ga–In–Sn ternary eutectic melting point of 10.7 ℃. When they changed the reaction temperature to make the alloys completely solid without liquid phase distribution, no hydrogen was produced. Interestingly, in many experiments, it was found that at 20–30 ℃, hydrogen generation from Al–Ga alloys stopped after only a certain extent [147, 149–153], but the reaction would resume if the system temperature was raised to resuscitate the liquid eutectic phase.

**Fig. 10:**
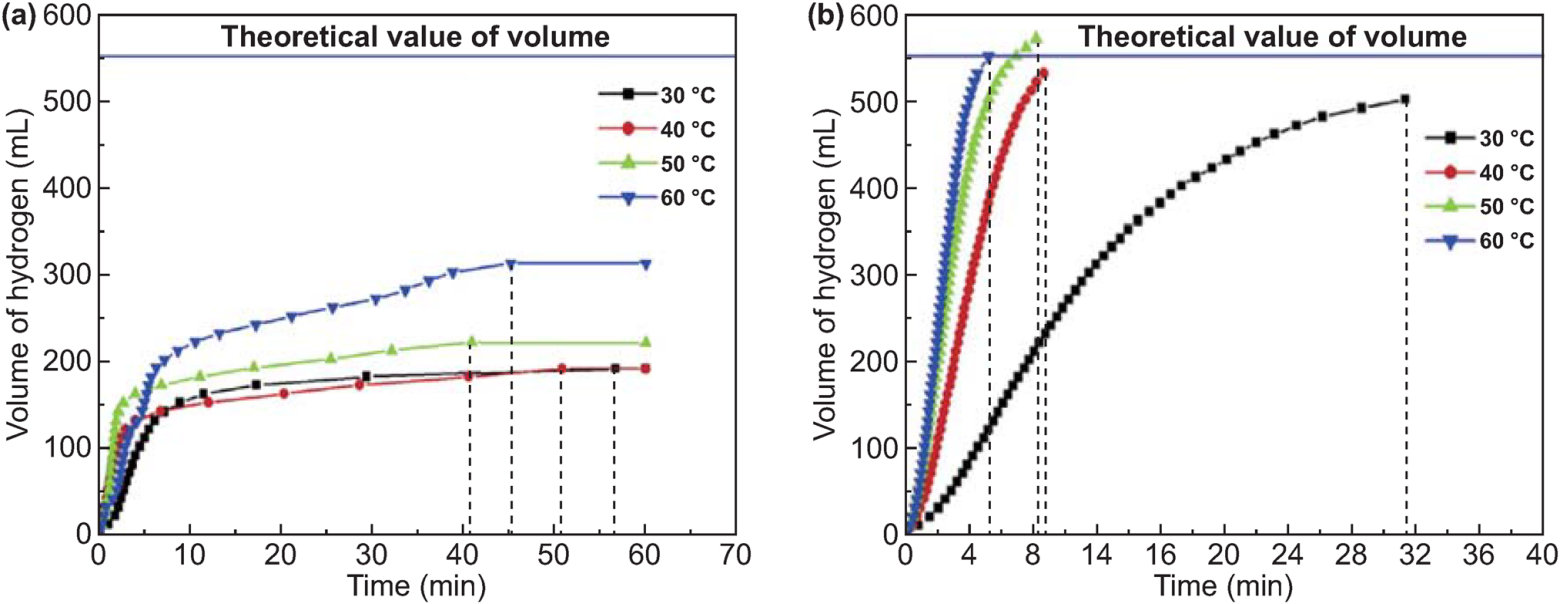
Water temperature effect on hydrogen generation of Al–Ga–InSn_4_ alloy and Al–Ga–In_3_Sn alloy (0.5 g alloy ingot in 100 mL water), **a** Al–Ga–InSn_4_ alloy and **b** Al–Ga–In_3_Sn alloy. Reprinted with permission from Ref. [150]. Copyright 2015 Elsevier

**Fig. 11:**
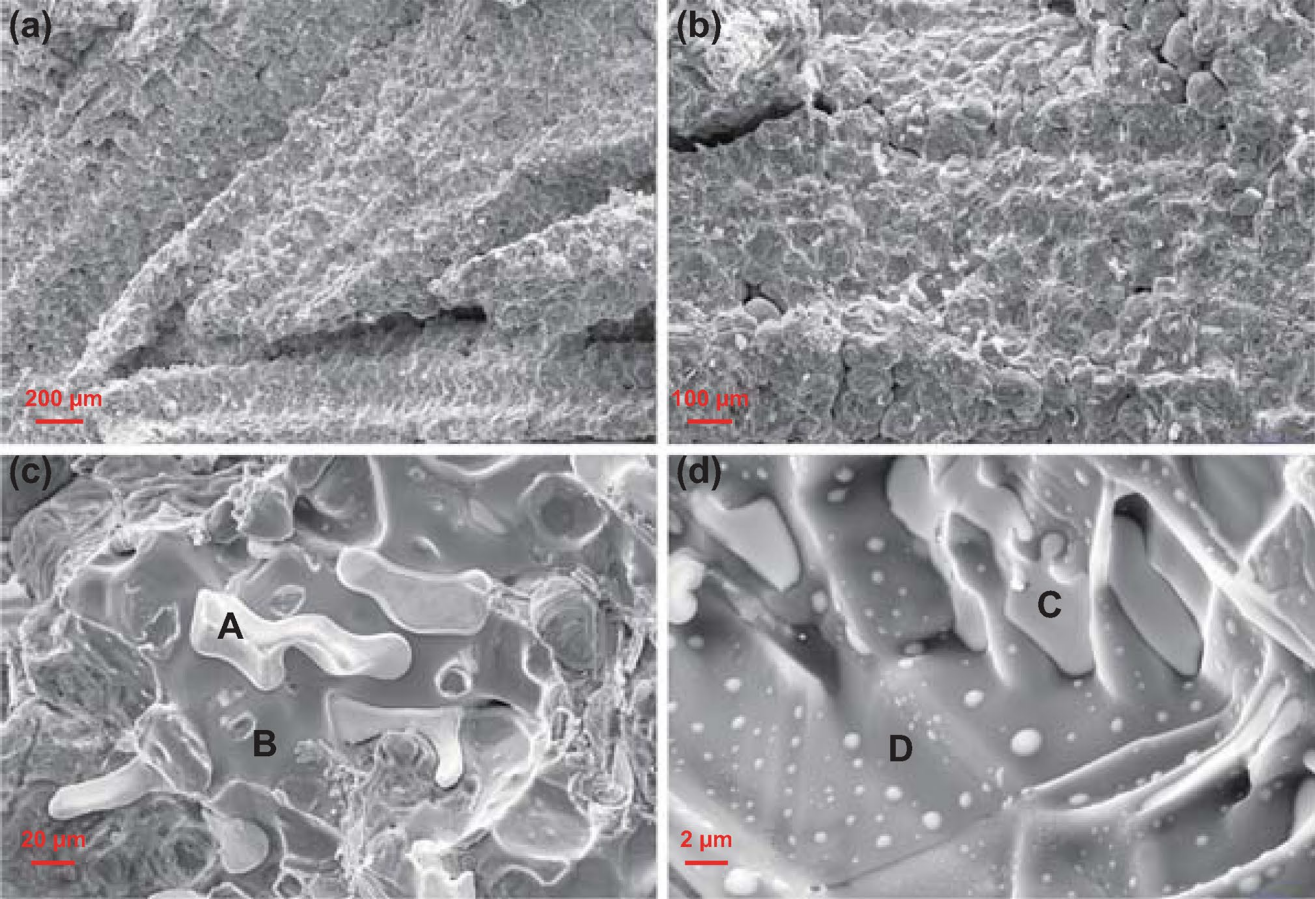
SEM images of fracture surfaces of Al 85 wt%–Ga_68.5_In_21.5_Sn_10_ alloy ingots, **a**, **b** images of the quaternary alloy, **c** enlarged image of intermetallic compounds, and **d** image of the LMPA at the grain boundary. Reprinted with permission from Ref. [155]. Copyright 2019 Elsevier

However, compared to the binary and ternary systems, the activity of the quaternary Al–Ga–In–Sn alloy was greatly improved and it could be fully reactive even at room temperature, indicating that the presence of a liquid eutectic phase in the Al-based alloy was essential. Liquid In_3_Sn and InSn_4_ were indeed observed in the Al–Ga–In–Sn quaternary system [154]. Qian Gao et al. [150] compared the hydrolysis properties of Al–Ga–InSn_4_ and Al–Ga–In_3_Sn alloys (Fig. 10). They concluded that the eutectic reaction of Al with InSn_4_ was crucial, and Al could transfer from Al grains to intermetallic compounds to react with water continuously. Recently, Lu et al. [155] investigated the hydrolysis performance and activation mechanism of Al 85wt%–Ga_68.5_In_21.5_Sn_10_ alloy (Fig. 11). Combined with EDX analysis, the marked regions in the SEM images shown in Fig. 11c, d could be identified as In_3_Sn phase (A), Al–Ga solid solution (matrix B), and C GaInSn liquid alloy (GIS) (C) and Al–Ga solid solution (matrix D). Especially, they emphasized the promotion of Al–water reaction with respect to the presence of low-melting eutectic liquid alloy GIS [156] and the In_3_Sn phase. The Al–water reaction can be summarized in two steps. Firstly, a certain amount of Al atoms, which are solvated in the GIS and In_3_Sn phases, are active and could react with the water freely. Secondly, the local temperature of the reaction site evidently increases due to a highly exothermic reaction, which can further promote the transportation of Al atoms to the interface and then react with water continuously.

**Fig. 12:**
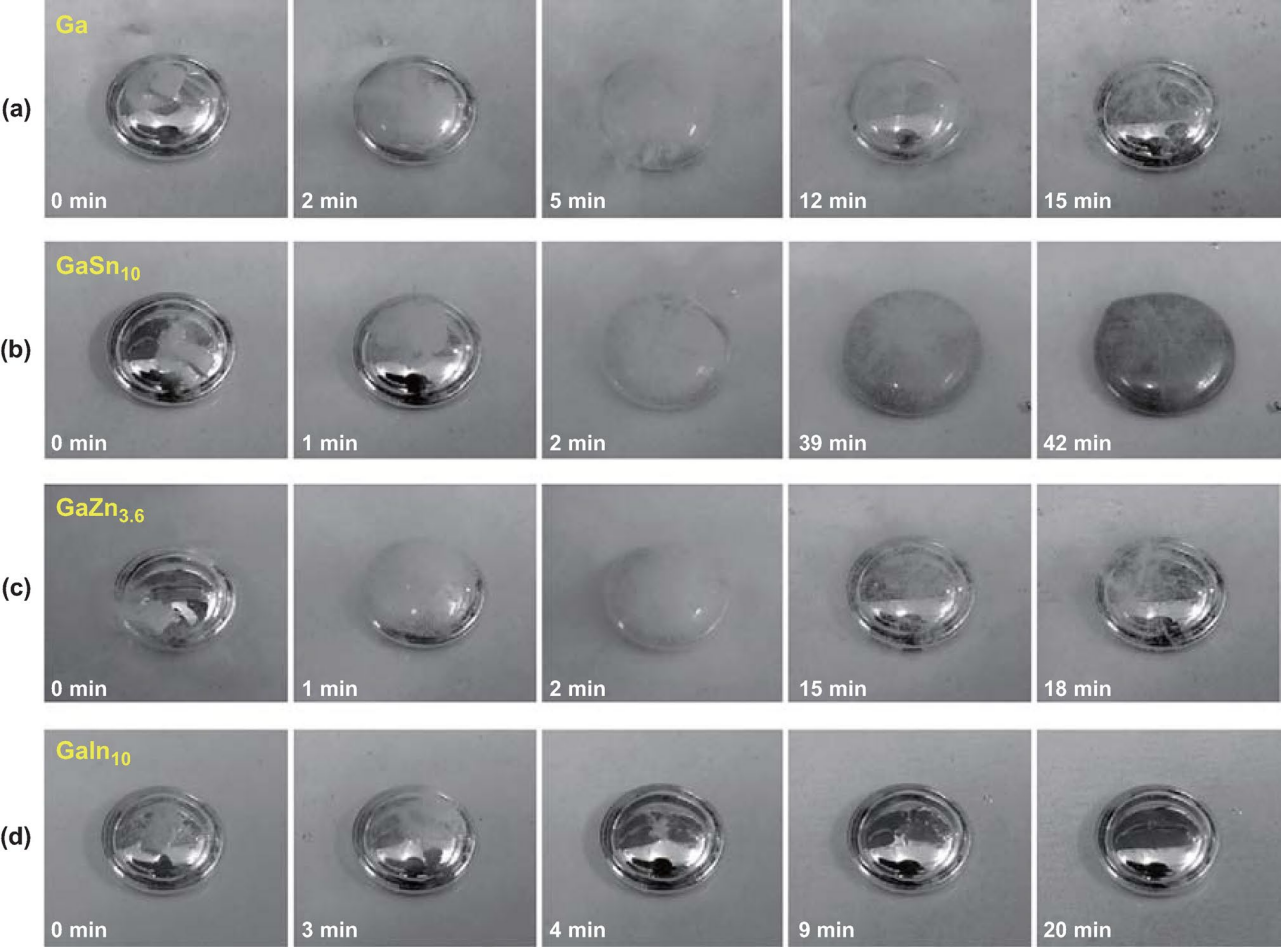
Surface morphology comparisons among different liquid metals in aluminum–water reaction. **a** Ga. **b** GaSn_10_. **c** GaZn_3.6_. **d** GaIn_10_. Reprinted with permission from Ref. [153]. Copyright 2016 Elsevier

It has been proven that alloying Al with low melting point metals is an effective approach to inhibit the formation of a coherent passivation layer and promote the hydrolysis kinetics. Liu et al. [153] tested Al on four different liquid alloys to produce hydrogen. It was found that aluminum completely dissolved in liquid GaIn_10_ in 4 min, and the liquid metal surface remained shiny, meaning that GaIn_10_ was stable during entire reaction process (Fig. 12). They designed pure Ga as a reactor and successively inlaid Al into it, and the process still achieved a great conversion yield after 5 times cycle without any dead-weight issues involved in system. Table 5 summarizes the varieties of some Al-based materials and their hydrolysis properties.

**Table 5:** Comparisons of some Al-based materials and their hydrolysis performances

Materials	Solution	Hydrogen yield (%)	mHGR (mL H_2_ (g min) ^−1^)	Activation energy (kJ mol^−1^)	Refs.
Al–15 wt% Ga_67_In_20.5_Sn_12.5_	Tap water	87.79% in 2 h	–	–	[155]
Al–10 wt% Ga_68_In_21_Sn_0.75_Bi_0.75_Zn_0.75_	Tap water	99.55% in 6 h	–	–	[155]
Al–10wt%Sn–5wt%Zn–5wt% MgH_2_	Pure water	72.6%	159.6	17.57	[157]
Al–10 mol% LiH–10 mol% KCl	Water	97.1%	1221.1	–	[157]
Al/Ni/*n*NaCl (Ni/Al = 2:10, *n* = 24 wt%) mixtures	Distilled water	92.9%	3.1	54	[158]
Al–16 wt% Bi alloy	1 M NaCl solution	92.75%	92	–	[159]
Aluminum with zinc amalgam activation	Water	–	–	43.4	[143]
Al–5wt%In–3wt%Zn–2wt%NaCl mixture	Water	82.9%	250	–	[160]
Al–Ga–OMC nanocomposite	Pure water	100%	112	–	[161]
50 wt%Al–34 wt%Ga–11 wt%In–5 wt%Sn	Distilled water	~ 83.8%	78	43.8	[162]
Al–Ga–In–Sn alloy	Water	–	~ 700	53 ± 4	[149]
Al–Ga–In–Sn–Fe(92.5:3.8:1.5:0.7:1.5) alloy	Distilled water	100%	120	–	[152]
1 ml liquid Ga + 50 mg aluminum block	NaOH solution	~ 88.7	~ 37.5	–	[153]
Al–3wt%Ga–3wt%In		–	180	–	[154]
Al–3wt%Ga–3wt%In–5wt%Sn	Water	99%	1080	–	[154]
Al–12Bi–7Zn (wt.%) powder	NaCl solution	98%	–	–	[163]
Al alloy/NaCl/1 g-g-C_3_N_4_	Tap water	94%	280	21.28	[164]
Al–10 wt%Li–5 wt%Sn	Water	100%	44.3	–	[165]
incomplete core/shell structures Al–20wt%Bi	Distilled water	83%	–	–	[166]
Al–7.5%Bi–2.5%In composite	Pure water	95.5%	194	–	[167]
Al–Ga–In–Sn alloy	Water (50℃)	95%	–	–	[168]
Al–30 wt%Bi–10 wt%C composites synthesized by high-pressure torsion	Pure water(60℃)	100%	270	–	[169]
Al–15 wt%NaMgH_3_-Bi-Li_3_AlH_6_	Distilled water	100%	1464	21.3	[170]
Al–10 wt%BiOCl–5 wt%LiH	Distilled water	94.9%	3178.5	26.9	[171]
(Al_2_Ga)‐8wt%In	Distilled water	70%	7.78	–	[172]
Al–1.0wt%Ga–1.5wt%In–3.0wt%SnCl_2_-1.0wt%Bi_2_O_3_ composite	Tap water	92%	1030.5	20.08	[173]
Al–Ga–In_3_Sn–Zn alloy	Deionized water	~ 95%	150	59	[174]
Al–Cu–Ga–In–Sn alloy	Distilled water	82.9%(50℃)	135	–	[175]
92Al–2 Mg–3.8 Ga–1.5In–0.7Sn	Distilled water	91%	14.8	–	[176, 177]
MHA–2%NaOH	0.5 M NaOH solution (55℃)	97.5%	421	29.3	[177]
Al with Graphite mixed Al(OH)_3_ (G-2) catalyst	Distilled water	100%	68	27.94	[178]
Al/Ni_0·1_/Cu_0·1_/H_2_O	Deionized water	70.6	96	–	[179]

### Hydrogen Production via Hydrolysis of Al-based Alloys or Its Hydrides

**Fig. 13:**
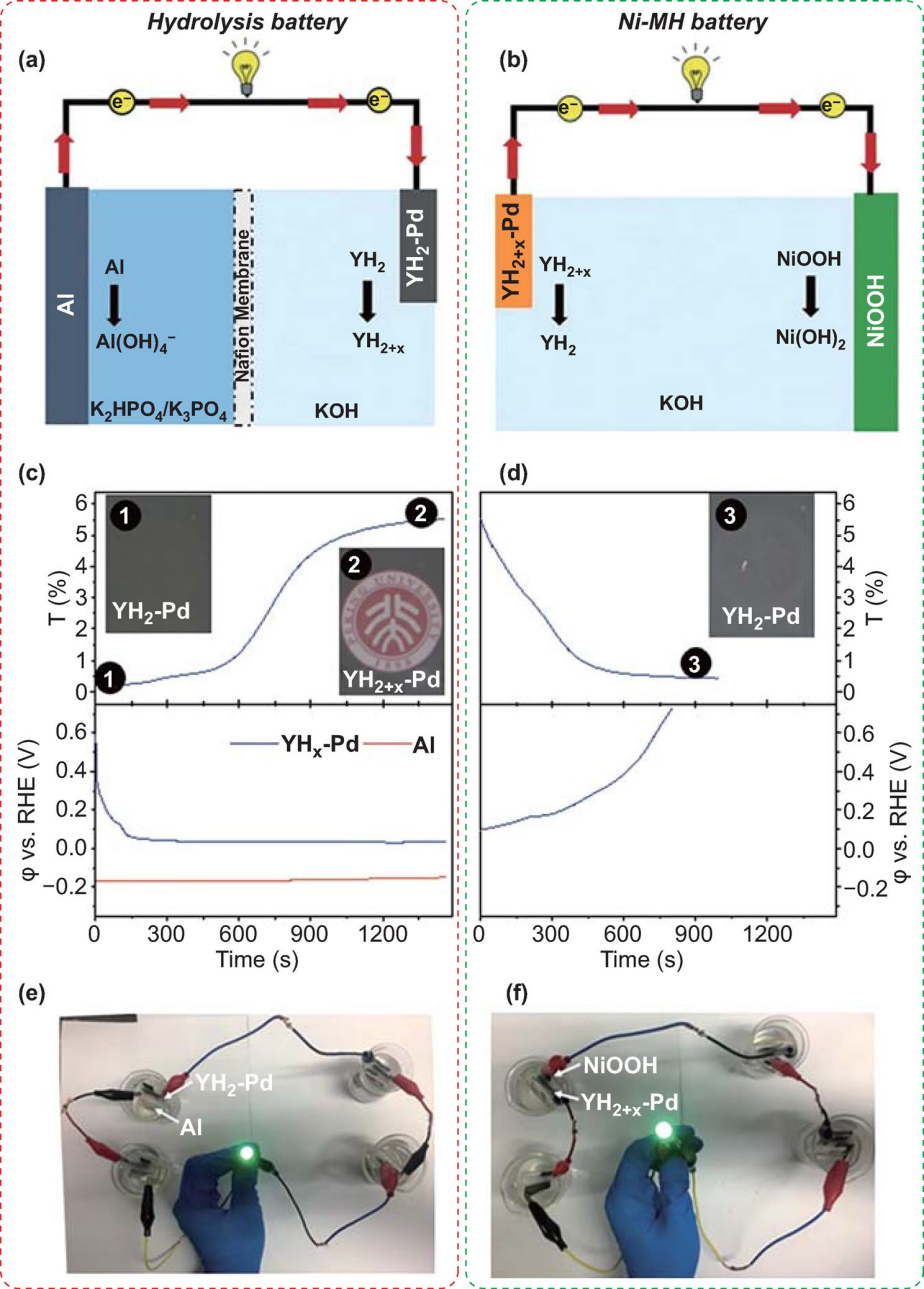
Schematic illustration of the Al hydrolysis battery **a** and the conventional Ni-MH battery **b** and their operation principle. Synchronized optical transmittance at 500 nm (the upper panel) and potential profiles (the lower panel) of the YH_x_-Pd electrode during galvanostatic process in the hydrolysis battery **c** and Ni-MH battery **d**. The current density is 0.2 and 0.05 mA cm^−2^ for the Al hydrolysis battery and the Ni-MH battery, respectively. The potential profile of the Al electrode during the operation of the hydrolysis battery is also shown. Inset: photographs of the YH_x_-Pd electrode at different stages as indicated by the corresponding number in the transmittance curve. Photographs of lighting the LED by the Al hydrolysis battery **e** and the MH-Ni battery (**f**). The electrolyte of hydrolysis battery and Ni-MH battery in **e**, **f** is 1 M KOH. Reprinted with permission from Ref. [181]. Copyright 2018 Wiley Online Library

Hydrolysis of metals or metal hydrides is a highly exothermic reaction; full hydrolysis of 1 mol aluminum generates 437 kJ heat and 1.5 mol hydrogen. An amount of 363 kJ energy can be produced unambiguously from this 1.5 mol hydrogen if it can be thoroughly utilized. Similarly, the hydrolysis of 1 mol magnesium generates 354 kJ heat and 1 mol hydrogen. While the exothermicity is huge during the metal–water hydrolysis, there were only few efforts that tried to transform the thermal energy into other forms of useful energy. In particular, Zhong et al. [180] calculated the energy efficiencies in the hydrolysis cycles of MgH_2_, H–Mg_3_La and H–La_2_Mg_17_. The maximum energy efficiencies of MgH_2_, H–Mg_3_La, and H–La_2_Mg_17_ were estimated to be 45.3%, 40.1%, and 41.1%, respectively, meaning roughly half of the energy released by the exothermic reaction was collected. Xiao et al. [181] firstly conceived and designed the Al-based hydrolysis battery, where the hydrolysis of Al was decoupled into a battery by pairing an Al foil with a hydrogen-storage electrode. In the hydrolysis battery, 8–15% of the hydrolysis heat was converted into usable electrical energy, leading to much higher energy efficiency compared to that of direct hydrolysis-H_2_ fuel cell approach. The schematic illustration of the hydrolysis battery is shown in Fig. 13, where the hydrolysis reaction of Al is a redox reaction. Thus, Al foil and a Pd-capped YH_2_ thin film were used as the anode and the cathode, respectively. As the hydrolysis battery was activated, the YH_2_-Pd electrode would convert into YH_2+x_ phase (x ≈ 1, the hydrogenated state), attaining the electrons flowed from Al. Desirably, the higher utilization of hydrolyzed thermal energy and more efficient kinetics controllability require further investigation.

## Recent Advances in Regeneration Process of Borohydrides from Hydrolysis By-products

It has been demonstrated that hydrogen supply from NaBH_4_ hydrolysis is a potential system for hydrogen generation. However, the hydrolysis reactions are plagued by irreversibility, and the resulting high-cost strikingly restrains the large-scale practical applications of these hydrolytic materials. Recently, Ouyang et al. developed a facile and economical method for NaBH_4_ regeneration by recycling its real-time hydrolysis products (NaBO_2_·2H_2_O and NaBO_2_·4H_2_O) for the first time without hydrides input [182, 183]. This may provide important insights for retrieving other hydrogen supply irreversible systems with high efficiency, such as LiBH_4_ or LiAlH_4_ production.

Recently, more attentions were shifted to the preparation and regeneration of NaBH_4_ for achieving its large-scale practical applications. In the industry of chemical production, NaBH_4_ is usually synthesized by the Brown–Schlesinger process [184] and the Bayer process [185]. The synthesis reactions of Schlesinger and Bayer methods are given as follows:

6 $$4{\text{NaH}} + {\text{B}}\left( {{\text{OCH}}_{3} } \right)_{3} \to {\text{NaBH}}_{4} + 3{\text{NaOCH}}_{3} \left( {225\sim275 \, ^\circ {\text{C}}} \right)$$

7 $${\text{Na}}_{2} {\text{B}}_{4} {\text{O}}_{7} + 16{\text{Na}} + 8{\text{H}}_{2} + 7{\text{SiO}}_{2} \to {\text{NaBH}}_{4} + 7{\text{Na}}_{2} {\text{SiO}}_{3} \left( {450\sim500 \, ^\circ {\text{C}}} \right)$$

Though the above technologies are mature, they are unsuitable for NaBH_4_ hydrolysis applications because of the fancy raw materials (Na or NaH) and high-energy consumption processes. Thus, suitable methods for NaBH_4_ synthesis have been developed with low-cost raw materials instead of sodium or its hydride. MgH_2_ was used to react with anhydrous borax (Na_2_B_4_O_7_) for NaBH_4_ synthesis by ball milling method at room temperature (RT). Here, the NaBH_4_ yield may reach 78% with the addition of Na_2_CO_3_ [186]. This method introduces not only a novel reducing agent (MgH_2_), but also an energy-efficient strategy for NaBH_4_ synthesis. Enlightened by this, RT ball milling became attractive in NaBH_4_ synthesis studies, by which Na and MgH_2_ could react with B_2_O_3_ with the NaBH_4_ yield of ~ 25% [187]. As Na was replaced by safe and cheap NaCl, NaBH_4_ could also be produced [188]. Subsequently, high-pressure milling was also developed to synthesize NaBH_4_. For instance, the synthesis of NaBH_4_ could be achieved by ball milling the hybrid of NaH and MgB_2_ under 120 bar H_2_ pressure with the yield of ca. 18% [189].

**Fig. 14:**
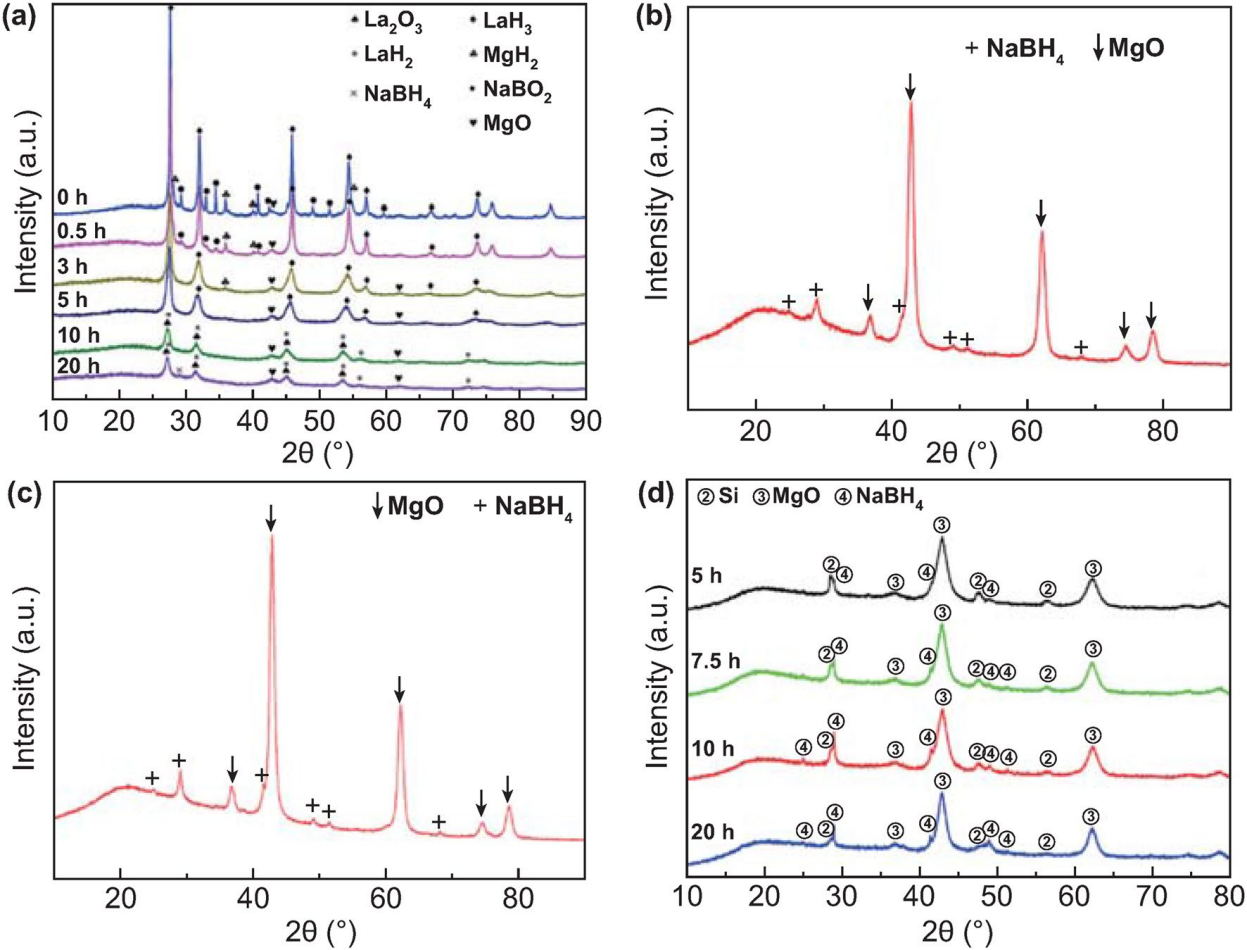
**a** XRD patterns of the NaBO_2_–Mg_3_La hydride hybrids and the product after ball milling the NaBO_2_-Mg_3_La hydride mixture. **b** XRD pattern of products via ball milling the mixture of NaBO_2_·2H_2_O-MgH_2_ in 1:5.5 mol ratio for 15 h. **c** XRD curve of products via ball milling the mixture of NaBO_2_·2H_2_O-5 Mg for 15 h. **d** XRD spectra of the products after ball milling Mg_2_Si and NaBO_2_·2H_2_O mixtures (in 2:1 mol ratio). Reprinted with permission from Ref. [198]. Copyright 2018 MDPI

**Fig. 15:**
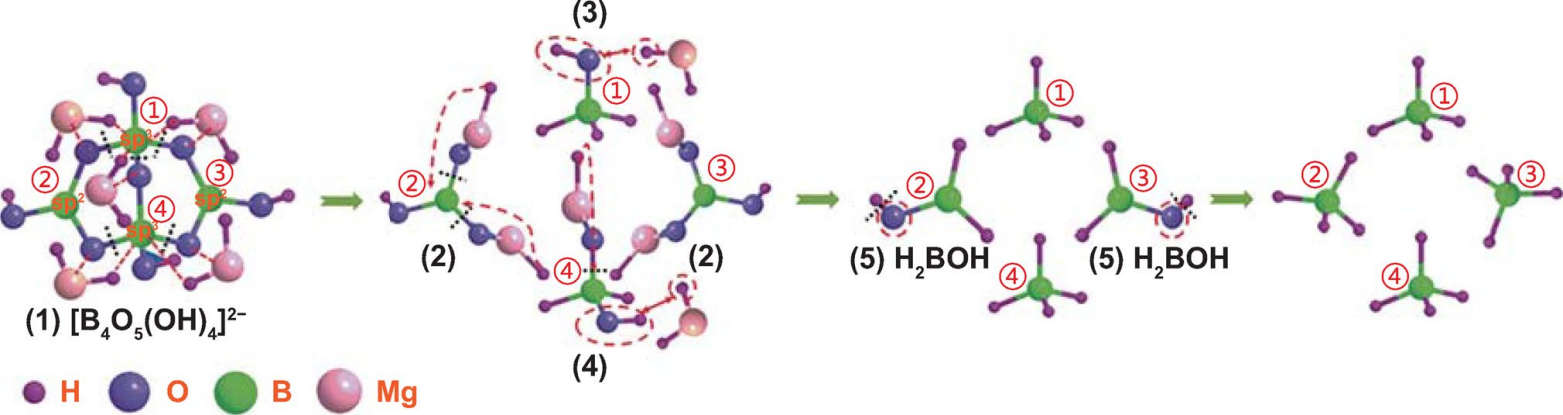
Proposed reaction mechanism between Mg, Na_2_CO_3_, and Na_2_B_4_O_7_·10H_2_O to form NaBH_4_. Reprinted with permission from Ref. [196]. Copyright 2020 Wiley Online Library

Importantly, considering the sustainability and environmental friendliness, NaBH_4_ regeneration from NaBO_2_·xH_2_O, the hydrolysis by-product, is appealing as the regeneration and hydrolysis form a recycling system. Since Kojima et al. [190] firstly achieved the regeneration of NaBH_4_ via reacting MgH_2_ with NaBO_2_ under 70 bar H_2_ pressure at 550 °C with a ~ 97% yield of NaBH_4_, NaBO_2_ has become the main research object for NaBH_4_ regeneration. Later, the thermochemistry process was substituted by RT ball milling because of high energy consumption under extreme conditions (high reaction temperature and high hydrogen pressure). Hsueh et al. [191–193] adopted MgH_2_ to react with anhydrous NaBO_2_ by ball milling under inert atmosphere. The conversion yields of NaBH_4_ were > 70%, which indicated that ball milling is advisable for the reaction between MgH_2_ and NaBO_2_. Recently, Ouyang et al. [182, 183, 194] successfully achieved the regeneration of NaBH_4_ (Fig. 14) by applying the real hydrolysis by-product (NaBO_2_·2H_2_O and NaBO_2_·4H_2_O) as raw material with Mg-based reducing agents (Mg, Mg_2_Si and Mg_17_Al_12_) at ambient conditions, where the troublesome heat-wasting process to obtain NaBO_2_ using a drying procedure at over 350 °C from NaBO_2_·xH_2_O was omitted. The regeneration yield of NaBH_4_ may reach 78%. Significantly, the charged H^−^ stored in NaBH_4_ was completely converted from protonic H^+^ in water bound to NaBO_2_. Particularly, it was found that the regeneration yield of NaBH_4_ was up ~ 90%, while MgH_2_ acted as reducing agent [195]. Recently, Ouyang et al. [196] found that high-energy ball milling of magnesium (Mg) with the mixture of Na_2_B_4_O_7_·xH_2_O (x = 5, 10) and Na_2_CO_3_ (obtained by exposing an aqueous solution of NaBO_2_ to CO_2_) resulted in the formation of NaBH_4_ with a high yield of 80% under ambient conditions. In their approach, after ball milling for just 10 min, only B_4_O_5_(OH)_4_^2−^ was detected (Fig. 15(1)), suggesting that the reaction started with this compound containing two BO_4_ tetrahedra and two BO_3_ triangles. The B–O bond with a bond length of 1.4418 Å in the BO_4_ tetrahedra is weaker than that (1.3683 Å) in the BO_3_ triangle. Thus, the B–O bond in the BO_4_ tetrahedra preferentially broke via a B–O–Mg–H intermediate, forming B–H and Mg–O (Fig. 15(2, 4)). In the following step, the cleavage of (B)–O–H (O bonded with *sp*^2^ boron) formed the H_2_BOH intermediate (Fig. 15(5)), in which B acted as the Lewis acidic site that accepted H^−^ from MgH_2_ leading to the formation of the final products, BH_4_^−^ and MgO. On the other hand, OH^−^ bonded with sp^3^ boron (Fig. 15(3, 4)) was also substituted by H^−^ from MgH_2_, forming BH_4_^−^. Furthermore, they achieved a higher yield of 93.1% for a short duration (3.5 h) by ball milling hydrated borax (Na_2_B_4_O_7_·10H_2_O and/or Na_2_B_4_O_7_·5H_2_O) with different reducing agents such as MgH_2_, Mg, and NaH under ambient conditions [197]. By replacing the majority of MgH_2_ with low-cost Mg, an attractive yield of 78.6% was obtained. These reactions occurred without extra hydrogen gas inputs, meaning the low-cost and sustainable regeneration. More detailed information toward NaBH_4_ regeneration can be found in a recent review [198].

In the past few years, numerous reports have been published dealing with the regeneration of NaBH_4_-based spent fuels (NaBO_2_·xH_2_O or Na_2_B_4_O_7_·xH_2_O), whereas the studies upon the regeneration of LiBH_4_-based spent products were quite limited. Bilen et al. [199] firstly utilized MgH_2_ and LiBO_2_ to synthesize LiBH_4_ by means of mechano-chemical reaction. Instead of its elements, the hydrolytic product of LiBH_4_ (LiBO_2_) was adopted as raw material, which may greatly reduce the application cost of LiBH_4_ by recycling spent products. However, the tricky heating-wasting process for obtaining anhydrous LiBO_2_ at elevated temperature (~ 470 ℃) is inevitable [200]. Stimulated by the successful regeneration of NaBH_4_, Ouyang et al. [201] reported a facile method to regenerate LiBH_4_ by ball milling its real hydrolysis by-product (LiBO_2_·2H_2_O) with Mg under ambient conditions with a yield of ~ 40%. This method bypasses the energy-intensive dehydration procedure to remove water from LiBO_2_·2H_2_O and does not require high-pressure H_2_ gas, therefore leading to much reduced costs. Interestingly, it is expected to effectively close the loop of LiBH_4_ regeneration and hydrolysis, enabling a wide deployment of LiBH_4_ for hydrogen storage and application. As same as NaBH_4_ or LiBH_4_, KBH_4_ could also be synthesized by mechano-chemical reaction. Bilen et al. [202] successfully synthesized KBH_4_ by ball milling KCl, MgH_2_, and B_2_O_3_ in a milling reactor. By tailoring the reactant ratio (MgH_2_/KCl) and the milling time, the yield of the reaction reached maximum values, whereas the definite value was not given.

Application of borohydride hydrolysis is limited by limit of their effective regeneration. Though the great achievements have been attained in the regeneration of NaBH_4_, simplifying synthetic routes and increasing regeneration yield that enable the efficient energy storage and conversion of the “one-pass” hydrogen fuel are two critical targets for large-scale applications. For the anhydrous NaBO_2_ recycling, it was found that MgH_2_ has the best reducing effect. However, its high cost, resulting from the high hydrogenation temperature of Mg, limits the application of such methods. For the direct NaBH_4_-based spent fuels (NaBO_2_·xH_2_O or Na_2_B_4_O_7_·xH_2_O), they can be reduced to NaBH_4_ with different reductants (MgH_2_, Mg, or Mg_2_Si) via ball milling, and the highest yield of NaBH_4_ may reach 93.1%. Moreover, this process, that uses hydrated metaborate or borax, bypasses the energy-intensive dehydration procedure to obtain anhydrous NaBO_2_ or Na_2_B_4_O_7_ without the requirement of high-pressure H_2_ gas; therefore, it could lead to much reduced costs. The boron compounds bound with water may act as hydrogen sources stored in NaBH_4_ instead of MgH_2_. As expected, low-cost waste Al or Al-based alloys may be attractive for achieving the regeneration of NaBH_4_ via ball milling, enabling a wide deployment of NaBH_4_ for hydrogen applications. This strategy may provide a new conceptual basis for the development of LiBH_4_ production or other borohydrides.

## Conclusions

The present review narrates the recent research progress of hydrogen generation via hydrolysis or alcoholysis by light metal-based materials for potential off- or on-board hydrogen applications, predominantly including borohydrides and Mg-/Al-based materials. The mechanisms of catalytic borohydride hydrolysis and activation of aluminum-based materials via alloying are depicted. Various common methods such as ball milling, catalysis, alloying, and solution modification for improving hydrolysis kinetics are described in detail. In summary, ball milling can refine the particles size to increase reaction activity, but it is unsuitable for practical use in the transportation and storage of the powder. For the hydrolysis of borohydrides, the Co–B-based materials are commonly considered as reactive as noble metals and much more cost-effective. Other metals and Co may form a synergistic effect in Co–B-based ternary or quaternary catalysts. The (catalyzed) hydrolysis of Mg-/Al-based materials has been summarized. The alcoholysis operated at low temperatures can supply hydrogen for special subzero circumstances. The cost is substantially decreased in regeneration of sodium borohydride, making hydrolysis/alcoholysis more practical for on-site hydrogen applications or fuel cells with the advantages of mild operating temperature, environmentally benign by-products, precise controllable of hydrogen release and high-purity H_2_. However, the major exothermicity of hydrolysis reactions has not received enough attention, which is even more than the hydrogen energy. The improvement of controllability of hydrolysis helps to design novel on-board hydrogen supply systems.
